# Predictors for a dementia gene mutation based on gene-panel next-generation sequencing of a large dementia referral series

**DOI:** 10.1038/s41380-018-0224-0

**Published:** 2018-10-02

**Authors:** C. Koriath, J. Kenny, G. Adamson, R. Druyeh, W. Taylor, J. Beck, L. Quinn, T. H. Mok, A. Dimitriadis, P. Norsworthy, N. Bass, J. Carter, Z. Walker, C. Kipps, E. Coulthard, J. M. Polke, M. Bernal-Quiros, N. Denning, R. Thomas, R. Raybould, J. Williams, C. J. Mummery, E. J. Wild, H. Houlden, S. J. Tabrizi, M. N. Rossor, H. Hummerich, J. D. Warren, J. B. Rowe, J. D. Rohrer, J. M. Schott, N. C. Fox, J. Collinge, S. Mead

**Affiliations:** 1 MRC Prion Unit at UCL, UCL Institute of Prion Diseases, Courtauld Building, London, W1W 7FF UK; 2 Department of Clinical Neurosciences, University of Cambridge, Cambridge, CB2 0SZ UK; 3 Medical Research Council Cognition and Brain Sciences Unit, Cambridge, CB2 7EF UK; 4 UCL Division of Psychiatry, Maple House, University College London, London, UK; 5 Essex Partnership University NHS Foundation Trust, Essex, SS11 7XX UK; 6 Wessex Neurological Centre, University Hospital Southampton NHS Foundation Trust, Southampton, UK; 7 Institute of Clinical Neuroscience, University of Bristol, Level 1 Learning and Research Building, Bristol, BS10 5NB UK; 8 Neurogenetics Laboratory, National Hospital for Neurology and Neurosurgery, Queen Square, London, WC1N 3BG UK; 9 Division of Psychological Medicine & Clinical Neurosciences, Cardiff University, Hadyn Ellis Building, Maindy Road, Cardiff, CF24 4HQ UK; 10 Dementia Research Centre, Department of Neurodegenerative Disease, UCL Institute of Neurology, Queen Square, London, WC1N 3BG UK; 11 Huntington’s Disease Centre, Department of Neurodegenerative Disease, UCL Institute of Neurology, Queen Square, London, WC1N 3BG UK

**Keywords:** Diagnostic markers, Psychiatric disorders

## Abstract

Next-generation genetic sequencing (NGS) technologies facilitate the screening of multiple genes linked to neurodegenerative dementia, but there are few reports about their use in clinical practice. Which patients would most profit from testing, and information on the likelihood of discovery of a causal variant in a clinical syndrome, are conspicuously absent from the literature, mostly for a lack of large-scale studies. We applied a validated NGS dementia panel to 3241 patients with dementia and healthy aged controls; 13,152 variants were classified by likelihood of pathogenicity. We identified 354 deleterious variants (DV, 12.6% of patients); 39 were novel DVs. Age at clinical onset, clinical syndrome and family history each strongly predict the likelihood of finding a DV, but healthcare setting and gender did not. DVs were frequently found in genes not usually associated with the clinical syndrome. Patients recruited from primary referral centres were compared with those seen at higher-level research centres and a national clinical neurogenetic laboratory; rates of discovery were comparable, making selection bias unlikely and the results generalisable to clinical practice. We estimated penetrance of DVs using large-scale online genomic population databases and found 71 with evidence of reduced penetrance. Two DVs in the same patient were found more frequently than expected. These data should provide a basis for more informed counselling and clinical decision making.

## Introduction

In recent years we have seen an increasing focus on research in dementia because of its rising prevalence in an aging society [1]. Although most dementias appear sporadic, familial forms of early-onset dementia with Mendelian inheritance (such as familial Alzheimer’s disease (AD), familial frontotemporal dementia (FTD) or inherited prion disease) have been crucial to furthering our understanding of the underlying clinical-pathological processes, and the ensuing development of animal models and experimental therapeutics [2]. Because of a series of high-profile failures of advanced clinical trials, clinical research has focussed on testing therapies earlier in disease using imaging and cerebrospinal fluid (CSF) biomarkers to support a pre-dementia diagnosis [1]. Clinical genetic studies offer the potential for presymptomatic diagnosis in at-risk individuals with a high degree of confidence about molecular pathology. Indeed, individuals carrying high-penetrance mutations may be the most appropriate groups in whom to test experimental therapeutics to prevent or delay neurodegeneration—especially if those therapeutics had been developed using animals expressing mutant human proteins [3].

Several factors have historically inhibited clinicians from considering a clinical genetic test in patients with dementia: lack of information about the probability of finding a high-penetrance mutation in single genes or the perception that this is unlikely; genetic heterogeneity (multiple genes causing the same pathology or clinical syndrome); high costs; the length of time to return results; and the lack of disease-modifying treatment options. These problems have been exacerbated recently because of a high rate of gene discovery and heterogeneity, particularly in FTD, with many genes not becoming available for clinical testing [4]. Furthermore, recent discoveries show marked pleiotropy, for example, the *C9orf72* expansion mutation being found in patients with clinically diagnosed FTD, amyotrophic lateral sclerosis (ALS), Huntington’s disease-like syndromes, and AD [5, 6]. The advent of next-generation genetic sequencing (NGS)-based gene-panel technology circumvents some of these problems by examining multiple genes simultaneously; however, reports of the use of panel diagnostics have been limited to small series that cannot provide the statistical power needed to support firm genetic evidence of pathogenicity of variants or clinical decision making [7].

In this multi-site retrospective and prospective study, we analysed a large series of samples from patients with heterogeneous dementia syndromes using a validated NGS panel for dementia [7] and using gold-standard processes and analytical strategies similar to those used by clinically accredited laboratories. The 17-gene panel was combined with amplification based assays of the *C9orf72* and *PRNP* expansion mutations, and exome sequencing in a large subset: therefore, we assessed all the known common causes, and most of the rarer causes of genetic dementia syndromes. We sought to establish statistically meaningful prevalences of genetically determined dementias in referred patients groups in order to provide data about predictive factors in the clinical assessment, rates of mutation detection in relevant mutation categories, mutations in genes unexpected for the phenotype, and multiple mutations in the same individual (concurrent). These analyses were performed on patient data classified by clinical diagnoses, not neuropathology, reflecting the real-life situations and uncertainty faced by clinicians. Using online large-scale sequencing and data sharing projects we also sought to clarify issues of causality and penetrance in the literature. These data may help in interpretation of variants and in formulating guidance about the clinical use of panel and genomic technologies in dementia.

## Methods

The study comprised 3241 samples: 2784 patient samples, with clinical rather than pathological diagnoses to reflect clinical reality at the point of care (1052 AD, 794 FTD, 299 prion disease, and 639 patients with a dementia syndrome not consistent with other categories and associated with motor symptoms (DemMot)), and 457 healthy elderly control samples. From 1998 to 2015 the UCL Department of Neurodegenerative Disease/MRC Prion Unit performed research genetic testing with clinical feedback for *PRNP*, *PSEN1*, *PSEN2*, *APP*, *GRN* and *C9orf72*. A total of 2352 UK patient samples were chosen retrospectively from these referred cases. Selection was based on the documentation of clinical parameters to be used in the predictive modelling and to equalise sample numbers in the different diagnostic, age, and family history categories and was blind to research data about the presence of a gene variant. Four hundred and thirty-two patient samples were referred prospectively for the study: 165 patients from National Health Service (NHS) cognitive disorders clinics in Southern England for gene-panel testing research, and 267 patient samples from the Division of Neurogenetics at the National Hospital for Neurology and Neurosurgery (NHNN) for clinical gene-panel testing. The study was approved by the local research ethics committee.

Clinical syndrome was based on the assessment of the referring physician at the time of referral. This included patients referred over a 20 year period; it would therefore not have been done according to a single standardised set of current research diagnostic criteria as these have been modified over this time. However, these changes in definition are unlikely to have led to misclassifications as we used only high-level clinical diagnostic categories, and changes in diagnostic criteria over time have principally focussed on achieving an earlier or biomarker supported diagnosis. DemMot was a category we defined to assemble a variety of clinical syndromes that comprise a cognitive disorder and pyramidal or extrapyramidal features, not fitting any of the other diagnostic categories, eg, Huntington’s disease-like (all were screened for the Huntington’s disease expansion), progressive supranuclear palsy, and corticobasal syndrome, to explore the usefulness of a dementia panel in cryptic movement disorder cases, which feature dementia as part of a complex syndrome. Prion disease patients were referred to the National Prion Clinic for *PRNP* gene testing based on a suspicion of inherited prion disease. FTD patients comprised the UCL and Cambridge University FTD Cohorts. CSF results were available to confirm the diagnosis in 78 cases from AD and FTD cohorts, and were consistent with clinical diagnosis in 91.0%. Neuropathological data were available for 122 patients (4.5% of all patients) and confirmed the clinical diagnosis in 102 (83.6%) of cases. Only one case with an unexpected mutation went to post-mortem examination (from our AD cohort with an unexpected *C9orf72* expansion). Neuropathological data confirmed the expected TDP pathology.

Age at clinical onset (AAO), gender, site of sample origin, and family history were documented from the clinical notes and referral cards. The strength of a patient’s family history was quantified with a modified Goldman score (GS) [8, 9], whereby GS1 corresponds to at least three affected family members over two generations linked by a first degree relative; GS2 relates to a patient from a family with three cases but not fulfilling the criteria for GS1. GS3 relates to one relative with early-onset dementia, or GS3.5 for one relative with late-onset dementia. Cases with a known negative family history were called GS4, whereas cases with a censored or unknown family history were categorised as GS4.5.

Additional to ethnicity documented in our database, we considered non-white British ethnicity by comparing genotypes at 133 sequenced SNPs from our study participants to those from individuals from British and continental outgroup populations genotyped by the 1000 Genomes study [10]. Although the number of SNPs used was small for inference of ancestry, we were able to identify population-specific clusters using principal components analysis implemented with PLINK [11], and therefore study participants who were outliers from a British cluster. In this way we identified 105 individuals (carrying 14 DVs out of 354 in total) with evidence of non-white British ethnicity (3.2%). DVs identified in these individuals did not bias overall findings; therefore we included these individuals in our reports of mutation frequency.

A total of 2974 samples (2517 patients, 457 controls) were run using the MRC Dementia Gene Panel on an IonTorrent PGM sequencer (Thermo Fisher Scientific), which had been previously validated in a blinded in-house study [7]. Similar gene panels using identical technologies are also in widespread use in clinically accredited laboratories [12]. For quality control and according to the protocol, target amplification was assessed via qPCR and enriched template-postive ion-sphere particles were measured on a Qubit® 2.0 Fluorometer. For each run, chip loading and the number of aligned reads were evaluated. The panel comprised the open reading frame and intron/exon boundaries of 17 dementia genes: *APP, CHMP2B, CSF1R, FUS, GRN, ITM2B, MAPT, NOTCH3, PRNP, PSEN1, PSEN2, SERPINI1, SQSTM1, TARDBP, TREM2, TYROBP* and *VCP*, and was supplemented by repeat-primed PCR assessment for *C9orf72* expansions [13], DNA size fractionation for the *PRNP* octapepide repeat insertional mutation [14], and *APOE* genotype by minor groove-binding probe. Data were aligned to the hg37 build in NextGENe, assessed for an at least 95% 10× target coverage and the VCF files exported to GeneticistAssistant (both Softgenetics) for further analysis. On average, samples had 157,350 mapped sequencing reads, 90% of which were on target. The average mean depth of coverage was 676 with an average uniformity of 94.7%; on average, 99.5% of the target sequence was covered at least 10-fold. In addition to the average coverage, coverage of the variant (>10×), zygosity and variant (allele) frequency were assessed for each variant being analysed in GeneticistAssistant; allele frequency should be between 0.25 and 0.75 for heterozygous variants, and between 0.8 and 1 for homozygous variants. If necessary, forward and backward reads (similar reads in both directions) were evaluated in NextGENe. *C9orf72* expansions and *PRNP* OPRIs were analysed in PeakScanner (LifeTechnologies). We screened the dataset for copy number variants using DeCon version 1.0.1[32], but could not validate any structural variants. Novel DVs were confirmed by Sanger-sequencing.

Two hundred and sixty-seven patient samples referred for testing to the Neurogenetics Laboratory at the NHNN were sequenced on an Illumina MiSeq or HiSeq platform using the Neurogenetics Laboratory Dementia Panel, which included 17 genes: *APP, CHMP2B, CSF1R, DNMT1, FUS, GRN, HTRA1, ITM2B, MAPT, NOTCH3, PRNP, PSEN1, PSEN2, TARDBP, TREM2, TYROBP* and *VCP*. Both the Neurogenetics Laboratory and the MRC Prion Unit team worked together on the validation of the original gene panel [7], both are laboratories experienced in the quality control, validation and clinical reporting of gene tests. For this study, only variants in genes overlapping the MRC Dementia Gene Panel were included. Library preparation and enrichment was performed using the Nextera Rapid Capture Custom Enrichment Kit (Illumina) according to manufacturer’s protocols. All RefSeq transcripts of the genes listed were targeted (coding exons ±15 bp flanking intronic sequences, with the exception of *MAPT*, which was sequenced to ±25 bp to cover known intronic splicing mutations). A minimum of 99% coverage at 30× and an average read depth of 500× was consistently obtained in samples; sequencing regions with coverage lower than 10× were manually inspected. DVs in these clinically sequenced samples were confirmed by bi-directional Sanger sequencing. These patient samples were not tested for *APOE*, *C9orf72* or *PRNP* insertional mutations.

Seven hundred and fifteen patients who were tested on the MRC Dementia Gene Panel (AD *n* = 509, FTD *n* = 83, DemMot = 31, Prion = 92, no controls) were also exome sequenced at Source Bioscience (Nottingham, UK). Agilent-based exome capture (Agilent, Santa Clara, US) was followed by paired-end sequencing on the HiSeq2000 sequencer (Illumina, San Diego, US). Sequencing reads were aligned to GRCh37 using Novoalign followed by QC and variant calling in the Genome Analysis Toolkit, and annotation with ANNOVAR. Mean coverage across the cohorts was 64×, and 81.5% of targeted bases were covered >10×. Two DVs were detected in genes not included in the 17-gene panel (see results). No other known pathogenic variants were returned by Ensembl’s Variant Effect Predictor [15] in the exome-sequencing data.

Variant classification followed the guidelines published by the American College of Medical Genetics and Genomics and the Association for Molecular Pathology in 2015 [16], for which we introduced clarifications specific to our disease circumstances and removed criteria unsuited to our setting (Table 1a and 1b). The algorithm used for classification is based on the level of evidence available for each variant (Table 1a), which is combined for a final classification (Table 1b). Intronic variants were assessed using Human Splicing Finder HSF V3.0 and classified according to our criteria [17]. Only variants with a population frequency <5% were manually classified. For the Neurogenetics Laboratory samples, we did not report likely benign, benign or synonymous variants.

**Table 1a:** Evidence used to classify variants according to their pathogenicity level

Evidence level	Criteria
Pathogenic Strong	1) Coding amino-acid change previously published as deleterious with evidence of segregation in more than one pedigree or in multiple unrelated patients with the same phenotype
	2) Null variant in a gene where loss of function (LOF) is a known disease mechanism (caveat LOF variants at extreme 3’-end)
	3) Variant in a gene associated with an expected very rare pathology (e.g., *PRNP* mutation and prion pathology)
	4) Explained mechanism of pathophysiology of variant using in vitro or in vivo studies
	5) Found in a mutational hotspot, i.e., a domain where many other pathogenic mutations are seen, generally with additionally support from in silico prediction software
Pathogenic Moderate	1) Coding amino-acid change previously and justifiably published as deleterious but without evidence of segregation or in a single pedigree/patient
	2) Novel missense change at an amino-acid residue where a different pathogenic missense change has been seen
	3) A very different amino-acid change at the same site or next to one with a less dramatic amino-acid change but deleterious
	4) In a gene, the mechanism of which is understood and the effect of the variant is in keeping with that mechanism
	5) Protein length changes as a result of in-frame deletions/insertions in a nonrepeat region or stop-loss variants
	6) Mutation in a gene associated with a rare pathology in a case with a compatible clinical syndrome
	7) Intronic variant affecting splicing or protein length
Pathogenic Supporting	1) Variant with a major amino-acid change near or in a functional domain (e.g., active site of an enzyme) but not in a mutational hotspot
	2) Multiple lines of computational evidence support a deleterious effect on the gene or gene product (conservation, evolutionary, splicing impact, etc.), caveat: because many in silico algorithms use the same or very similar input for their predictions, each algorithm should not be counted as an independent criterion
	3) Reported in both cases and controls, but more cases than controls (statistically significant in a study)
Pathogenic Risk Factor	1) Previously reported as risk factor, either variant itself or clear established pattern in gene
	2) > 1 in 10000 in *gnomAD*
	3) The prevalence of the variant in affected individuals is significantly increased compared with the prevalence in controls
Benign Independent	Allele frequency > 5% on *gnomAD*, or 1000 genomes project
Benign Strong	1) Allele frequency > 1% on *gnomAD*
	2) Reported benign in multiple pedigrees or with insight into gene/protein mechanism
	3) Allele frequency is greater than expected for disorder
	4) Lack of segregation in affected members of a family, caveat: phenocopies and penetrance
	5) Seen in equal or greater frequencies in controls than cases
Benign Moderate	1) Allele frequency over 0.1% on *gnomAD*
	2) Reported benign in one case or pedigree
	3) Genetic mechanism inconsistent with pathological phenotype, or known mutation spectrum
Benign Supporting	1) Missense variant in a gene for which primarily truncating variants are known to cause disease or the mechanism is very specific and known
	2) Multiple lines of computational evidence suggest no impact on gene or gene product (conservation, evolutionary, splicing impact, etc.)
	3) A synonymous (silent) variant for which splicing prediction algorithms predict no impact to the splice consensus sequence

Variants identified in a sample were classified according to the information available about them. This included the type of mutation in question, its position in the gene and/or protein, its frequency in online population databases, in silico predictions of effects on proteins, and whether it had previously been reported in families, single cases or controls

**Table 1b:** Criteria for variant classification

Pathogenicity	Algorithm
Deleterious	Found in patient(s) and not controls OR in significant excess in patients AND seen on *gnomAD* at < 1 in 50,000; AND Pathogenic Strong evidence 1) OR 2), PLUS one additional Pathogenic Strong or two Pathogenic Moderate or one Pathogenic Moderate and one Pathogenic Supporting criterion
Likely deleterious	The prevalence of the variant in affected individuals is significantly increased compared with the prevalence in controls, or only seen on *gnomAD* at < 1 in 10,000; AND Pathogenic Moderate evidence 1) OR 2) OR 3) AND one additional Pathogenic Strong or Moderate or Supporting criteria
Possibly deleterious	Found on *gnomAD* at < 1 in 5000 and at least one Supporting criterion
Uncertain	Insufficient or conflicting evidence Missense mutation not nearby other missense mutations thought to be pathogenic
Likely benign	One Benign Strong criteria OR one Benign Moderate AND one Benign Supporting criteria OR two Benign Supporting criteria
Benign	Benign Independent OR one Benign Strong evidence criterion AND two further Benign Moderate or Benign Supporting criteria
Risk factor	Previously reported as risk factor, either variant itself or clear established pattern in gene, AND > 1 in 10,000 in *gnomAD*; AND the prevalence of the variant in affected individuals is significantly increased compared with the prevalence in controls

The evidence available about each variant was combined to determine its likely effect and likelihood of causing disease

Statistical analysis for associations, predictors and relative risks were performed in SPSS (IBM, Version 24) and included logistic regression, univariate analysis of variance (ANOVA), and contingency tables. Statistical analyses were carried out with a pre-defined statistical threshold of *p* < 0.01 to account for testing five key independent hypotheses (see tests labelled Ϯ); subsequent secondary, exploratory tests were carried out without further corrections for multiple testing.

Penetrance calculations were based on estimates of lifetime risk generated using a Boolean literature search of PubMed for 'dementia' AND 'epidemiology' from 2008 to January 2017 to determine incidence and prevalence of early-onset AD (EOAD) and early-onset FTD (EOFTD). Subsequent calculations were performed in Microsoft Excel based on the methodology used in prion disease [18], both for variants identified in this data set and reportedly pathogenic variants described in the literature (see Supplementary data for more details).

## Results

Baseline characteristics of patients and controls are shown in Table 2.

**Table 2:** Baseline characteristics of the included patient cohorts and controls

Cohort	Total *N*	Male (%)	Mean AAO	Early onset (%)	Goldman score						Site of sample origin				
					1	2	3	3.5	4	4.5	NHNN cognitive centre	NHS cognitive disorder clinics	National prion clinic	NHNN Clinical neurogenetics	Cardiff university
AD	1052	400 (38.0%)	57.7	78	73 (6.9%)	30 (2.9%)	66 (6.3%)	103 (9.8%)	245 (23.3%)	535 (50.9%)	873 (83.0%)	103 (9.8%)	1 (0.1%)	75 (7.1%)	0
FTD	794	419 (52.8%)	58.4	61	72 (9.1%)	43 (5.4%)	50 (6.3%)	64 (8.1%)	298 (37.5%)	267 (33.6%)	645 (81.2%)	45 (5.7%)	0	104 (13.1%)	0
Prion	299	121 (40.5%)	57.6	55	34 (11.4%)	6 (2.0%)	11 (3.7%)	15 (5.0%)	207 (69.2%)	26 (8.7%)	2 (0.7%)	0	296 (99.0%)	1 (0.3%)	0
DemMot	639	280 (43.8%)	55.8	70	24 (3.8%)	12 (1.9%)	18 (2.8%)	84 (13.2%)	215 (33.7%)	286 (44.8%)	535 (83.7%)	17 (2.7%)	0	87 (13.6%)	0
Controls	457	237 (51.9%)	76.6	–	4 (0.9%)	0	0	2 (0.4%)	0	451 (98.7%)	6 (1.3%)	0	3 (0.7%)	0	448 (98.0%)
All	3241	1457 (45.0%)	60.3	59	207 (6.4%)	91 (2.8%)	145 (4.5%)	268 (8.3%)	965 (29.8%)	1565 (48.3%)	2061 (63.6%)	165 (5.1%)	300 (9.3%)	267 (8.2%)	448 (13.8%)

The 3241 samples were made up by 1052 AD patients, 794 FTD patients, 299 prion patients and 639 patients with dementia with motor symptoms, as well as 457 elderly controls. This table shows the distribution of each cohort in numbers and percentages for their baseline characteristics sex, age at onset (AAO), early- or late-onset disease (over or under 65 AAO), Goldman score [30] and the site of the sample origin, i.e., whether it was a retrospective sample chosen from the collection of a cognitive centre, a prospectively recruited sample from a Memory Clinic at a primary referral centre, a sample from the National Prion Clinic or a control sample

### Classification of variants

**Fig. 1:**
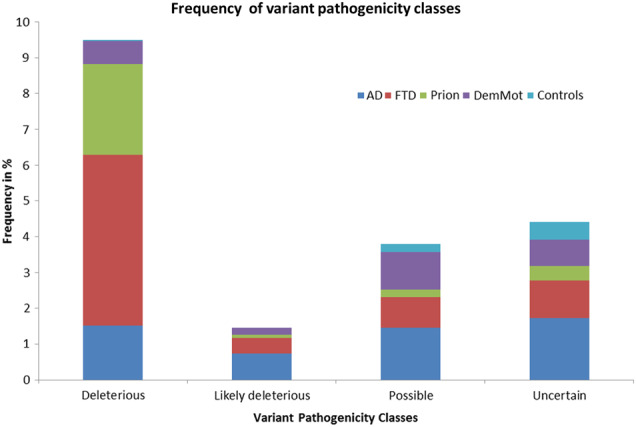
Frequency of variant pathogenicity classes in the data set. This figure shows the frequency of the various variant pathogenicity classes in the total data set broken down by individual phenotypes

There is no computational or experimental tool to perfectly classify individual variants by their pathogenicity, the current start-of-the-art clinical method is decision making that considers multiple factors and is adaptable to multiple potential genes/disease mechanisms (Table 1a and 1b). In this way we classified 13,152 variants in 3241 individuals (Fig. 1, Tables 2, 3) and identified 352 DVs (deleterious or likely deleterious variants) in 341 patients (12.2% of patients, *p* = 2.8 × 10^−14^, OR: 31.8, 95% CI (7.88, 127.94)). Two additional DVs were seen in two of 457 controls (0.4%, Fig. 1, Table 3). In addition to these 343 individuals carrying DVs, 121 possibly deleterious variants were found in 3.5% of all samples (4.1% patients, 1.3% of controls), in excess in cases vs. controls (*p* = 0.005 (Ϯ), OR: 3.06, 95% CI (1.34, 7.01)), suggesting that two-thirds of variants in this category might be reclassifiable as DVs if sufficient data were available; these warrant further research specific to each case including the potential for segregation in families. One hundred and forty-three variants that could not be classified as benign or deleterious, termed uncertain, were seen in 4.4% samples (4.6% patients, 3.5% controls, *p* = 0.39).

**Table 3:** Number of variants in each pathogenicity class observed in the present data set

N variants		Deleterious	Likely deleterious	Novel DVs	Possible	Uncertain	Likely benign	Benign	Risk Factor	Synonymous	Total N cohort
Early-Onset	AD	38 (4.6%)	19 (2.3%)	12 (1.5%)	34 (4.2%)	70 (8.6%)	39 (4.8%)	545 (66.6%)	147 (18%)	1957 (239.2%)	818
	FTD	112 (23%)	10 (2.1%)	14 (2.9%)	13 (2.7%)	65 (13.3%)	24 (4.9%)	314 (64.5%)	81 (16.6%)	1364 (280.1%)	487
	Prion	57 (35%)	1 (0.6%)	0 (0%)	4 (2.5%)	2 (1.2%)	5 (3.1%)	93 (57.1%)	29 (17.8%)	563 (345.4%)	163
	DemMot	19 (4.3%)	4 (0.9%)	2 (0.4%)	14 (3.1%)	20 (4.5%)	24 (5.4%)	278 (62.2%)	82 (18.3%)	1221 (273.2%)	447
	Controls	0 (0%)	0 (0%)	0 (0%)	0 (0%)	0 (0%)	47 (1175%)	5 (125%)	2 (50%)	17 (425%)	4
	Total	226 (11.8%)	34 (1.8%)	29 (1.5%)	65 (3.4%)	157 (8.2%)	139 (7.2%)	1234 (64.3%)	341 (17.8%)	5122 (266.9%)	1919
Late-Onset	AD	9 (4%)	4 (1.8%)	3 (1.3%)	12 (5.3%)	9 (4%)	91 (40.3%)	166 (73.5%)	39 (17.3%)	698 (308.8%)	226
	FTD	16 (9.9%)	2 (1.2%)	3 (1.9%)	6 (3.7%)	20 (12.3%)	59 (36.4%)	84 (51.9%)	34 (21%)	451 (278.4%)	162
	Prion	5 (7.5%)	1 (1.5%)	0 (0%)	2 (3%)	1 (1.5%)	17 (25.4%)	29 (43.3%)	21 (31.3%)	204 (304.5%)	67
	DemMot	2 (1.1%)	1 (0.6%)	0 (0%)	8 (4.5%)	6 (3.4%)	85 (48.3%)	100 (56.8%)	17 (9.7%)	471 (267.6%)	176
	Controls	2 (0.4%)	0 (0%)	0 (0%)	5 (1.1%)	12 (2.7%)	3 (0.7%)	314 (70.1%)	49 (10.9%)	1415 (315.8%)	448
	Total	34 (3.2%)	8 (0.7%)	6 (0.6%)	33 (3.1%)	48 (4.4%)	254 (23.5%)	690 (63.9%)	160 (14.8%)	3239 (300.2%)	1079
All Ages	AD	48 (4.6%)	23 (2.2%)	15 (1.4%)	46 (4.4%)	79 (7.5%)	130 (12.4%)	717 (68.2%)	187 (17.8%)	2676 (254.4%)	1052
	FTD	155 (19.5%)	14 (1.8%)	20 (2.5%)	24 (3%)	107 (13.5%)	99 (12.5%)	500 (63%)	132 (16.6%)	2262 (284.9%)	794
	Prion	82 (27.4%)	3 (1%)	1 (0.3%)	6 (2%)	6 (2%)	35 (11.7%)	162 (54.2%)	67 (22.4%)	999 (334.1%)	299
	DemMot	22 (3.4%)	5 (0.8%)	3 (0.5%)	23 (3.6%)	32 (5%)	119 (18.6%)	387 (60.6%)	106 (16.6%)	1750 (273.9%)	639
	Controls	2 (0.4%)	0 (0%)	0 (0%)	5 (1.1%)	12 (2.6%)	51 (11.2%)	321 (70.2%)	54 (11.8%)	1447 (316.6%)	457
	Total (% of patients)	309 (9.5%)	45 (1.4%)	39 (1.2%)	104 (3.2%)	236 (7.3%)	435 (13.4%)	2087 (64.4%)	546 (16.8%)	9134 (281.8%)	3241

The total number of variants in each variant pathogenicity class identified in each of the respective cohorts is shown as well as a percentage of the number of cases in each cohort for those of uncertain or at least possible pathogenicity. In 243 patient and control samples we could not be certain of age at onset, therefore the sum of Early and Late-Onset does not equal All Ages. A DV was identified in two healthy elderly control samples; one elderly male carried the *PRNP* Gln212Pro variant and one elderly male was found to carry a frameshift mutation in *GRN* (c.708 + 5_708 + 8delGTGA) affecting a splice-site.

**Fig. 2:**
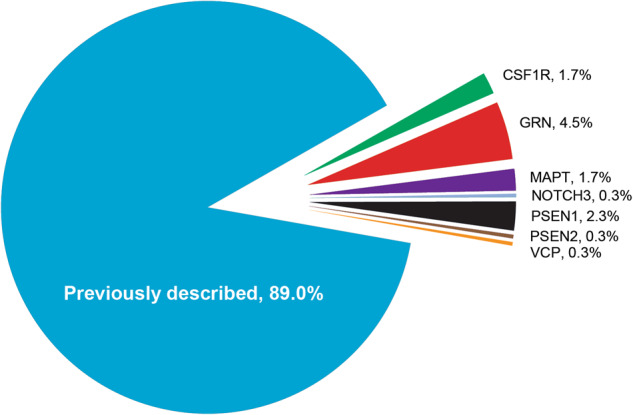
Genes in which novel DVs were found. Eleven percent of DVs were not previously described in the literature (*n* = 39). Known mutations were found in *PRNP* (24.6%), *C9orf72* (19.2%), *MAPT* (15.8%), *GRN* (9.9%), *PSEN1* (9.6%), *APP* (3.4%), *CSF1R* (2.0%), *VCP* (1.7%), *SQSTM1* (0.9%), *TARDBP* (0.6%) and *CHMP2B*, *ITM2B*, *NOTCH3, PSEN2* and *TREM2* (0.3% each)

Novel variants were defined as variants that had not previously been reported in the literature nor found in the Genome Aggregation Database (*gnomAD*) of exomes and genomes [19]. Out of the 343 samples with 352 DVs detected in this data set, 39 (11.3% patients with DVs, *χ*^2^ test *p* = 0.004 (Ϯ)) were novel DVs, Fig. 2; 16 were identified in *GRN*, eight in *PSEN1*, six in *MAPT*, six in *CSF1R* and one each in *NOTCH3*, *PSEN2* and *VCP*. Because of the well-known disease mechanism of *GRN* related to loss of function, novel variants in this gene were easier to classify as DVs than those in genes with less well-understood pathomechanisms.

There have been several reports of concurrent pathogenic mutations in patients with FTD [20–22]. Eleven patients were found to carry two DVs (out of chance expectation, *p* < 0.001 (Ϯ), Binomial test [23], 3.2% of patients with at least one DV; one AD patient, seven FTD patients, two patients with prion disease and one patient with DemMot). There was a notable excess in FTD, as previously suggested by case reports (observed 7, expected 2.7, *χ*^2^ test, *p* = 0.003) [20].

Fourteen DVs were observed in 12 patient samples of non-UK ancestry; of these, two were *C9orf72* expansions and two cases carried a double DV. None of the DVs found in samples of non-UK ancestry were observed on *gnomAD*, except for the secondary variant of one of the concurrent mutations, which was described in four South Asian, one East Asian and three European samples, with a frequency of 0.013%, 0.0058% and 0.0027%, respectively.

Rare GRN missense variants found in cases and controls were all classified as possibly pathogenic. Collectively, compared with controls (1.1%), *GRN* missense variants were seen significantly in excess in patients (3.6%, *p* = 0.004, OR 3.4 (1.4–8.4)) with AD (3.5%, *p* = 0.006, OR = 3.3) and FTD (3.7%, *p* = 0.006, OR = 3.4) but not DemMot (2.5%, *p* = 0.1, OR = 2.3) or prion disease (2.7%, *p* = 0.2, OR = 2.5). Compared with controls (3.7%), heterozygous *TREM2* missense variants were not significantly more common in dementia syndromes (4.9%; *p* = 0.3), or in AD alone (5.9%, *p* = 0.097; OR = 1.6), albeit consistent with effect sizes previously reported [24].

715/2984 samples that were analysed with the NGS gene-panel were also exome sequenced (see Methods), which allowed discovery of only two additional mutations classified as DVs, in *TBK1* [25] and *DNMT1* [26], and no DVs in other neurology-relevant genes not included in the panel.

All the analyses reported here were repeated following exclusion of known family members (*n* = 73), on the basis of the proband being identified in the family history of the second case, with no significant change to any finding.

### Phenotypes associated with deleterious or likely deleterious variants

Neurodegenerative disease syndromes caused by DVs were found to have broadly similar ages at onset (AAO), independent of the gene in which these variants were identified (Supplementary Figure 1). An overall statistically significant difference in AAO by gene (ANOVA, *p* = 0.003) was driven by the relatively late clinical onset in *GRN* and the relatively early onset of patients with DVs in *PSEN1*, *MAPT* and *PRNP*. Nevertheless, patients with *PRNP* DVs presented with a very wide range of AAO, stretching into old age (range 22–79 years). DVs discovered in old age were not restricted to *PRNP*, and included *APP*, *PSEN1*, *C9orf72*, *GRN*, *MAPT*, *CHMP2B*, *CSF1R*, *TYROBP* and *VCP*.

**Fig. 3:**
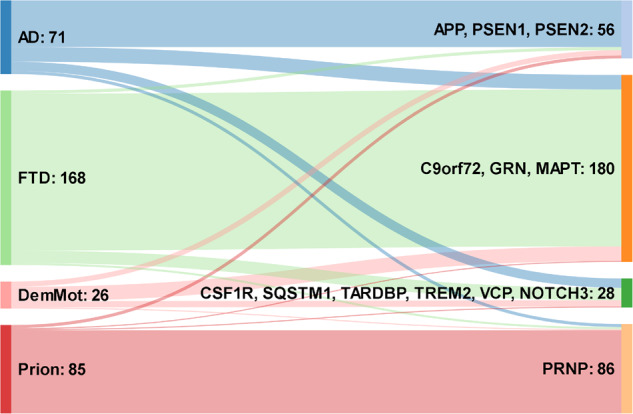
Chart illustrating the association between clinical syndrome and gene implicated. Numbers on the left refer to patients with clinical syndromes, numbers of the right refer to DVs in implicated genes

DVs were often discovered in patients with clinical syndromes that would not normally prompt a request for sequencing of the implicated gene. In 58 patient samples, DVs were identified in genes that would not normally be screened in the clinical syndrome (16.9% of all patients with DVs, *p* = 0.013 (Ϯ)), Fig. 3. In patients diagnosed with AD we found three *C9orf72* expansions, nine DVs in *MAPT*, five in *CSF1R*, two in *GRN*, three in *PRNP* and one each in *SQSTM1*, *TARDBP* and *VCP*, as well as a homozygous *TREM2* DV normally associated with Nasu-Hakola disease. For FTD patients, five were seen in *VCP*, three variants each were seen in *CSF1R* and *PSEN1*, two in *PRNP* and SQSTM1, and one each in *NOTCH3* and *CHMP2B*. Two DVs in *PSEN1* and one each in *GRN* and *VCP* were identified in patients referred with suspected prion disease, the latter as part of a concurrent mutation with a *PRNP* DV. In DemMot, four DVs were identified in *PSEN1*, three variants in *MAPT*, as well as one variant each in *ITM2B*, *PRNP*, *GRN* and *PSEN2* were found.

### Predictors of deleterious or likely deleterious variants (Ϯ)

Coverage, sex, ethnicity, healthcare setting, and prospective/retrospective recruitment did not influence the likelihood of a DV (*p* = 0.97, *p* = 0.33, 0.68, 0.61 and *p* = 0.53, respectively, logistic regression); we therefore combined genders, ethnicities, sample coverage, sampling method and sample referral sites in further analyses. Compared with controls, AD (logistic regression, *p* = 0.006; Odds ratio (OR): 7.46, 95% confidence interval (CI) (1.77, 31.49)), FTD (*p* = 2.0 × 10^−6^; OR: 33.58, 95% CI (7.95, 141.81)), Prion patients (*p* = 2.24 × 10^−9^; OR: 92.54, 95% CI (20.98, 408.184)) and DemMot patients (*p* = 0.042; Odds ratio: 4.7, 95% CI (1.06, 20.87)) were significantly more likely to carry a DV in order of declining frequency Prion > FTD > AD > DemMot (Table 3).

**Fig. 4:**
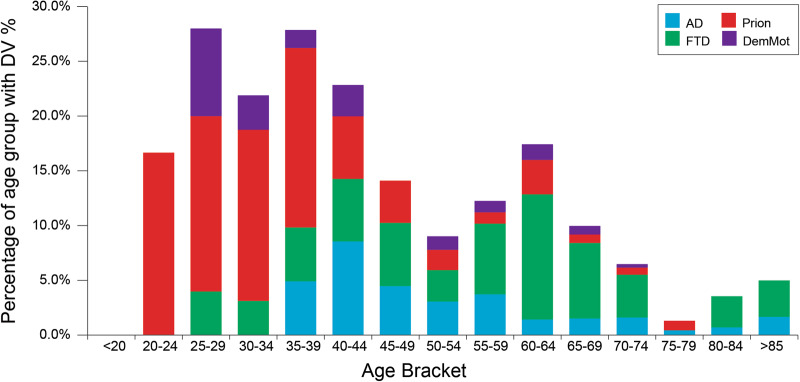
Proportion of patients with a deleterious or likely deleterious variant per age group (%). The distribution is skewed towards the younger ages of onset, but we discovered many patients with DVs associated with elderly ages of onset, particularly in the presence of a family history

**Fig. 5:**
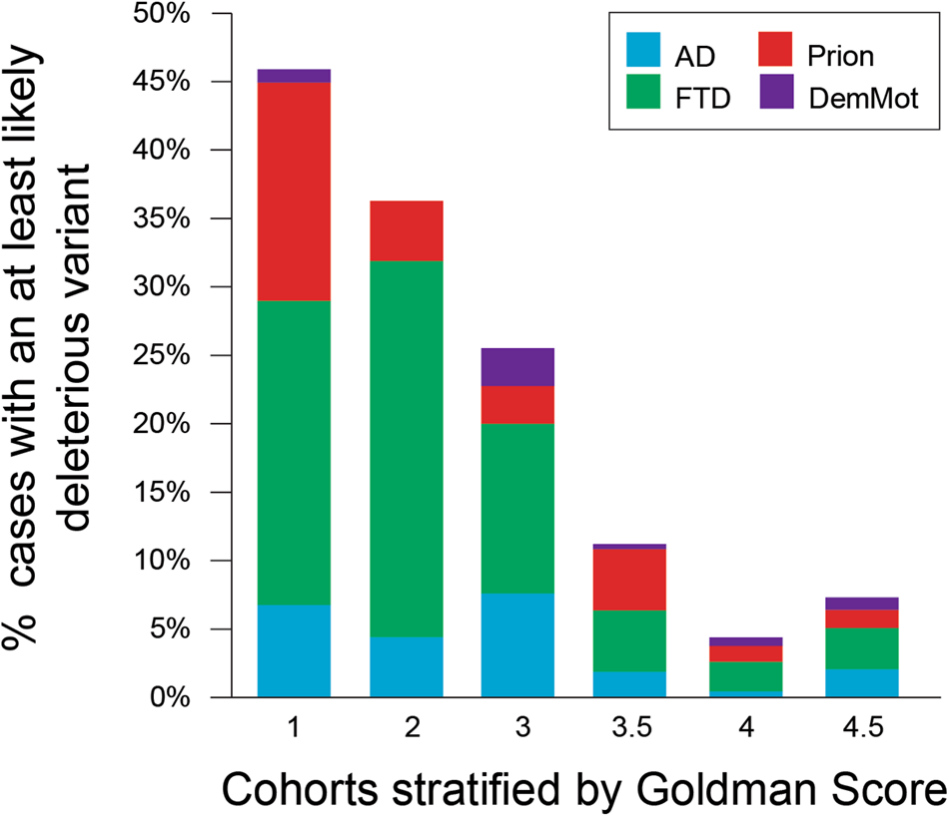
Family history is a strong predictor for the identification of a deleterious or likely deleterious variant Stratifying cases by Goldman Score reveals its strong predictive value in identifying cases with a DV; however, deleterious or likely deleterious variants are found in clinically relevant proportions of cases with no (GS4) or a censored (GS4.5) family history

**Fig. 6:**
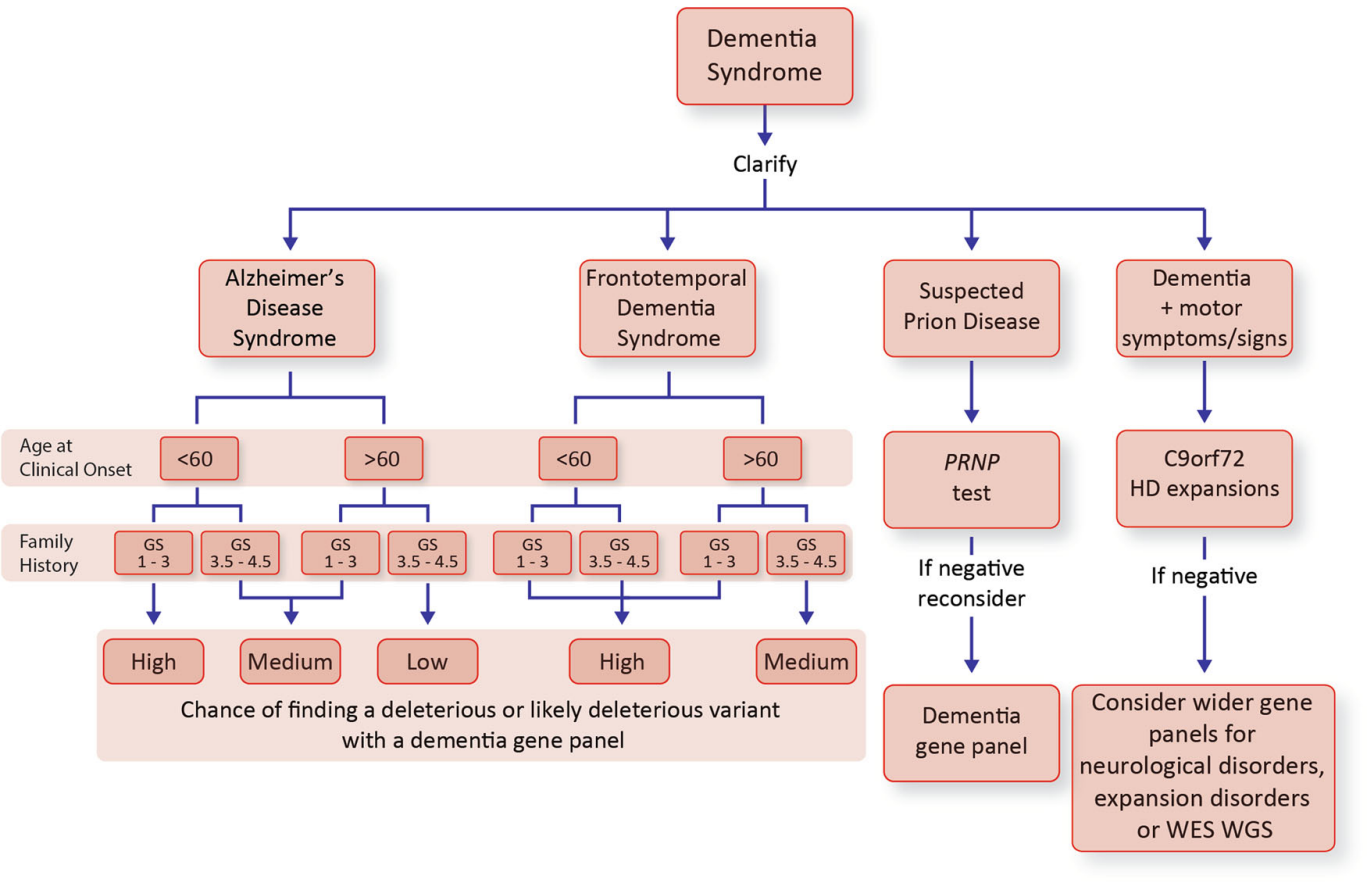
Suggested decision making about use of dementia gene-panel testing. We found gene-panel diagnostics was most useful in AD and FTD syndromes where it was hard to predict the implicated single gene. Yield of clinically relevant mutations was high (> 10%), medium (5–10%) or low (< 5%) in groups stratified by age and family history. The decision to refer for gene-panel diagnostics is not solely driven by the chance of a clinically relevant result, and many clinicians would consider even a low yield (< 5%) justifies use of a gene-panel in many clinical scenarios. A decision should take into consideration the wishes of the patient and at-risk individuals. FTD subtype (behavioural variant, progressive non-fluent aphasia, semantic dementia, etc.) may also influence the approach to testing but this requires further study. Suspected prion disease patients are best referred for *PRNP* testing in the first instance, and if this is negative, reconsider as per AD syndrome. Dementia-motor syndromes had a generally low yield on dementia panel testing (< 5% all subgroups), recommendations have been made for the stepwise investigation of HD-like syndromes, which are often caused by expansion disorders not well ascertained by gene-panel diagnostics [5]

AAO was a very strong predictor of finding a DV (*p* = 3.8 × 10^−9^, logistic regression, Fig. 4). Risk was high from early adulthood through to middle age and steadily declined into old age without clear change in risk at the traditional boundary of early and late-onset disease, age 65. Family history was also highly predictive of a DV (*p* = 4.6 × 10^−38^, logistic regression, Fig. 5). This association was also strong in late-onset dementia, in which circumstance GS remained highly predictive of identifying a DV (*p* = 2.3 × 10^−4^, logistic regression), but age at onset no longer had a significant effect (*p* = 0.452). The combined effects of AAO, clinical syndrome and family history were considered in recommendations for use of dementia gene panels (Fig. 6).

In the AD cohort, unsurprisingly, *ApoE* genotypes 3/4 and 4/4 were significantly enriched (*p* = 3.0 × 10^−4^ and *p* = 0.001, *χ*^2^ test, respectively); for FTD patients, the *ApoE* genotype 4/4 was also significantly enriched (*p* = 6.7 × 10^−4^, *χ*^2^ test). In total, 75% of GS1 AD patients and 71% of GS1 FTD patients (all ages) had either a DV or one *ApoE4* genotype.

### Penetrance calculations in EOAD and EOFTD

Minikel et al. [18] evaluated the penetrance of *PRNP* variants showing that some of those previously suspected to be DVs were neither highly penetrant nor benign. We therefore attempted to discover similarly partially penetrant DVs using the gene panel. The method used by Minikel requires an estimate of lifetime risk of disease and population frequency of a variant. We estimated lifetime risk of EOAD (1 in 3194) and EOFTD (1 in 3276) (see Supplementary data). We assessed the likely penetrance of reported DVs in *APP*, *PSEN1* and *PSEN2* for EOAD and *GRN*, *MAPT* and *VCP* for EOFTD both in the literature and novel variants discovered in this study. Assuming a proportion of autosomal dominant genetic cases of 10% for EOAD [27] and 20% for EOFTD [28], our calculations led to an expectation of 26 DVs among the 141,352 individuals on the *gnomAD* online database [19], but instead 182 variants were counted. For EOFTD, we would have expected to see 51 DVs in *gnomAD*, whereas we found 36 variant counts . The prevalence of AD deleterious variants in the literature [29] therefore vastly exceeds our estimates based on the prevalence of genetic early-onset dementia, in the assumption of high penetrance and high ascertainment. We therefore went on to test whether evidence from our study and data publicly available would revise classification of variants and clarify those deemed 'unclear'.

In the molgen database [29] and on the mutation database of Alzforum, 302 variants in *APP*, *PSEN1* and *PSEN2* and 153 variants in *GRN*, *MAPT* and *VCP* were listed as deleterious and a further 21 variants in *APP*, *PSEN1* and *PSEN2* and 64 variants in *GRN*, *MAPT* and *VCP* were reported as having unclear pathogenicity (Supplementary data). Many of the reported DVs are not observed at all, or found at very low frequencies resulting in estimates of penetrance with very wide confidence intervals, however, 71 purported DVs were detected in *gnomAD*, calling into question the extent of their pathogenicity and penetrance (Supplementary Tables 1S–3S). Based on their relative frequency in cases and the general population, most of the variants classified as DVs in our data set appear to be highly penetrant pathogenic mutations, with three exceptions. *APP* Ala713Thr was observed once in our AD cohort and despite being reported as pathogenic in the literature, its population frequency suggests low penetrance for EOAD of 0.4% (95% CI 0.1–2.4%), *MAPT* Gly389Arg (observed twice in our data set) was estimated to have 10.2% penetrance (95% CI 1.6–63.5%) and *PSEN1* Ile227Val (observed once in our data set) was estimated to have 2.9% penetrance (95% CI 0.3–26.9%) (See Tables 1S–3S for more details).

## Discussion

Numerous Mendelian genetic causes of dementia have been discovered over the last 28 years, but the translation of this knowledge into routine clinical practice has been limited [30]. Until recently, only a small number of tests were clinically available. Here, we try to bridge this gap by providing data to support gene-panel diagnostics in dementia through analysis of a series of patients enriched for those likely to be carrying deleterious mutations, and large enough to inform clinical practice. The identification of clinically relevant variants in our series was high in all groups aside from the elderly with a negative family history and those with dementia and motor symptoms that may not be caused by variants in typical dementia genes. We also discovered a high rate of novel variants and known variants in genes that even an experienced clinician would probably not have selected for single gene tests based on the clinical syndrome. Our results therefore justify broader clinical testing than hitherto customary. We identified clinical syndrome, age and the strength of the family history as predictive factors that should help guide counselling and decisions about referral for testing. Although many variants of uncertain significance remain and additional evidence is needed, these data, in tandem with large-scale population data, provide some of the evidence base needed for improved information and guidance in genetic testing.

Clinical syndrome was a strong predictor of the chance of detecting a mutation and the gene, but in markedly different ways. Ninety-four percent of suspected prion disease cases with DVs were linked to a single gene, *PRNP*; 93.5% of FTD patients with DVs were linked to three major, and two additional genes associated with FTD syndromes (*C9orf72, GRN, MAPT, SQSTM1, VCP*); however, in only 63% of clinically diagnosed AD patients were the DVs in genes linked to AD pathologies (*APP*, *PSEN1* or *PSEN2*). DVs in patients with a dementia-motor syndrome were uncommon and heterogeneous in their associations. These findings have implications for clinical practice: it would be reasonable to refer suspected prion disease patients for testing of *PRNP* alone. For FTD and AD syndromes, the dementia gene-panel approach seems sensible owing to the diversity of genes involved and phenotypic heterogeneity. Dementia-motor syndromes are more challenging however; a low rate of DV discovery either implies that disease relevant variants are not covered by our panel. This would not be surprising as we did not screen genes associated with familial Parkinson’s disease or the expansion disorders linked to HD phenocopy syndromes other than *C9orf72*; alternatively, these patients may harbour a low rate of single gene disorders. Despite the prominent role dementia plays in these patients’ clinical syndromes, a panel covering typical dementia genes only is of limited use in this cohort; more research is needed to resolve this question.

The low additional rate of mutation detection by research exome sequencing argues in favour of panel-based testing, which should be more cost-effective and avoids issues related to incidental detection of clinically relevant variants. We have not generated data to allow a rigorous comparison of alternative gene-panel technologies or composition, augmented exomes, genomes or other diagnostic approaches.

Age at onset was also a strong predictor of finding a DV. However, this was not an absolute rule, with the rate of DV detection being 13.5% in those with AAO < 65 and 7.2% in those with AAO > 65, which was a surprising finding perhaps related to the selection bias inherent in our referral based sample. Family history remained an important predictor in all age groups. Our findings therefore encourage the use of gene panels in late-onset dementia where there is evidence of a family history measured using a tool like the Goldman score. Only 6 DVs in 233 patients (2.6%) were found in late-onset dementia with a negative family history (GS4), three in *PRNP*, two *C9orf72* expansions and one in *GRN*, so if the family history is negative in late-onset dementia it seems reasonable not to consider gene-panel testing, as would be normal practice at the moment. We recommend dementia gene-panel diagnostics are considered in all early-onset patients, and late-onset patients with evidence of a genetic disorder in the family history (GS1, 2 or 3) [11]. We have made suggestions based on these findings (see flowchart Fig. 6); beyond the report of our experience, opinions will vary among physicians and patients about what level of risk justifies gene-panel testing. Indeed, some clinicians/patients/families may feel that even low risks < 5% of a DV would justify testing.

As there are no proven disease-modifying treatments yet available, some may question why an effort should be made to identify Mendelian causes of dementia [31]. However, many arguments can be brought in favour of identifying patients who carry pathogenic variants. These include the provision of a precise diagnosis, removing the need for further potentially invasive diagnostic tests and providing information about prognosis. It also opens up access to patient support from a community with a shared molecular pathology, who often lend strong lobbying and practical support to research and care in their condition. Genetic diagnosis allows for precision medicine in current and forthcoming clinical trials, for which there are several active examples. A positive genetic test in a relative also provides the opportunity for siblings or descendants to make an informed decision about testing. Our data should provide information to help doctors and genetic counsellors discuss risks with patients and their families to make informed joint decisions about clinical gene testing.

Our sampling strategy was to be representative of cases being considered for genetic testing by referring physicians. Bias in referrals owing to selection will have influenced the prevalence of DVs seen in this study vs. a population-based study or unselected dementia patients; however, a true population study would have to be very large indeed to detect a similar number of DVs. Although acknowledging selection bias as a limitation, we found no difference in rates of DV detection in prospectively referred cases, or those referred for a UK NHS clinical accredited service, implying that our findings are generalisable to cognitive clinic cases that physicians might consider referring. We were also limited by a selection of dementia genes and blinded validation studies that were done in 2014. The only major Mendelian disease gene to be discovered in the study’s relevant dementia syndromes is *TBK1* associated with FTD (found in a single patient by exome sequencing).

More work is needed to improve information in the literature and databases about the pathogenicity and penetrance of variants. An excess of potential DVs is seen in population data, incompatible with the observed prevalences of early-onset dementias. Both for EOAD and EOFTD, variants have been reported as potentially deleterious, which are most likely either benign or low penetrance. Large-scale studies harnessing the power of NGS are vital tools to ensure clinical diagnosis, testing and feedback are as accurate as possible. Improved sharing of patient genetic data, the availability of large-scale population data, improved in silico and in vitro modelling, particularly for less commonly involved genes and dementia syndromes should help improve the accuracy of classification [19]. We encourage the development of guidelines and funding to support sharing of clinical and genetic data in databases to further improve the accuracy of classification.

Increased genetic testing of a wide range of patients with diverse dementia syndromes promises opportunities for the patient, clinician and research, but also implies a burden for Clinical Genetics services. Similarly, predictive genetic testing in blood relatives ensuing from diagnosis in a proband, can have a considerable psychological impacts and the involvement of at-risk individuals from families in decisions about gene diagnostics is crucial.

## Electronic supplementary material

Supplementary Material
