# Supplementary material for: Predictors for a dementia gene mutation based on gene-panel next-generation sequencing of a large dementia referral series
Source: Mol Psychiatry. 2018 Oct 2;25(12):3399–412. doi: 10.1038/s41380-018-0224-0 (PMC6330090; doi:10.1038/s41380-018-0224-0)
Supplement: Supplementary file 1 — Supplementary Material [file 41380_2018_224_MOESM1_ESM.docx]

**Supplementary Material to Panel-based next-generation gene sequencing of a large dementia referral series**

C Koriath^1,9^, J Kenny^2^, G Adamson^2^, R Druyeh^2^, W Taylor^2^, J Beck^2^, TH Mok^2^, A Dimitriadis^2^, P Norsworthy^2^, N Bass^3^, J Carter^3^, Z Walker^3^, C Kipps^4^, E Coulthard^5^, JM Polke^6^, M Bernal-Quiros^6^, N Denning^7^, R Thomas^7^, R Raybould^7^, J Williams^7^, CJ Mummery^8^, EJ Wild^9^, H Houlden^6^, SJ Tabrizi^9^, MN Rossor^8^, H Hummerich^2^, JD Warren^8^, J B Rowe^10^, JD Rohrer^8^, JM Schott^8^, NC Fox^8^, J Collinge^2^, S Mead^2*^

[Prevalence of early-onset Alzheimer’s disease 2](#_Toc505256677)

[Prevalence of early-onset FTD 2](#_Toc505256678)

[Risk of early-onset Alzheimer’s disease 2](#_Toc505256679)

[Risk of early-onset Frontotemporal dementia 2](#_Toc505256680)

[Summary and cross-check with national mortality statistics 2](#_Toc505256681)

[Penetrance of genetic variants linked to early-onset Alzheimer’s disease and early-onset fronto-temporal dementia 3](#_Toc505256682)

[Published reportedly pathogenic variants 3](#_Toc505256683)

[Variants detected in the present dataset 4](#_Toc505256684)

[Figure 1S: Age at clinical onset (AAO) in patients with a DV, per gene 5](#_Toc505256685)

[Figure 2S: Frequency of variant pathogenicity classes in the dataset 6](#_Toc505256686)

[Figure 3S: Suggested decision making about use of dementia gene panel testing 7](#_Toc505256687)

[Table 1S: Reported pathogenic variants in Alzheimer’s Disease genes *APP*, *PSEN1* and *PSEN2* 8](#_Toc505256688)

[Table 2S: Reportedly pathogenic variants in Frontotemporal Dementia genes *GRN*, *MAPT* and *VCP* 9](#_Toc505256689)

[Table 3S: Penetrance of variants observed in our own dataset in *APP, PSEN1, PSEN2, GRN, MAPT* and *VCP* 10](#_Toc505256690)

[Table 4S: Tabulation of novel DVs 12](#_Toc505256691)

[Table 5Sa: Evidence used to classify variants according to their pathogenicity level 17](#_Toc505256692)

[Table 5Sb: Criteria for variant classification 17](#_Toc505256693)

[Table 6S: Number of variants in each pathogenicity class observed in the present dataset 18](#_Toc505256694)

# Prevalence of early-onset Alzheimer’s disease

Lambert *et al.*(1) offer an overview of the prevalence of early-onset AD. In the nine studies reviewed by Lambert *et al.*, prevalences for EOAD range from 10.6 (age at onset 20-64 years) to 200 (age at onset 55-64 years) per 100,000. Several groups determine prevalences between 20 and 40 for the age at onset range 45-64 years. Some variation in estimates may be a reflection of different methodologies and age ranges in each of these studies as the prevalence of AD varies greatly with age. We chose a prevalence estimate for EOAD of 30 per 100,000.

###

# Prevalence of early-onset FTD

Luukkainen *et al.* (2) present a summary report of the prevalence of early-onset FTD (EOFTD) as well as their own findings from northern Finland. Coyle-Gilchrist *et al.* looked at the prevalence of FTD in two UK counties (3). While methodology and analysed age groups varied between the different analyses, the reported prevalence for EOFTD ranged from 2.7 to 35 per 100,000. Overall, we chose a prevalence of 18 per 100,000 cases for EOFTD.

##

# Risk of early-onset Alzheimer’s disease

Based on our estimates of prevalence, we calculated the lifetime risk of developing either EOAD or EOFTD (age of onset between 30 and under 65 years of age).

Given the prevalence EOAD of approx. 30/100,000 and the average disease duration for EOAD of approx. 13 years (4), new cases per year are estimated at 2.31/100,000. Based on the population aged 30 to 64 of England and Wales from the 2015 dataset (22,435,600 people), new cases per year are estimated at 518. New cases through a 35 year window for this population of England and Wales are expected at 18,121. For the population of England and Wales in 2015 (57,885,400 people), this would amount to a ratio of 0.00031 or 1 in 3,194 people will develop EOAD in their lifetime.

###

# Risk of early-onset Frontotemporal dementia

For EOFTD, the prevalence is approx. 18/100,000 and disease duration is approx. 8 years on average(5). New cases per year are therefore estimated to occur at a rate of 2.25/100000, or 504 cases for the population aged 30 to 64 of England and Wales from the 2015 dataset (22,435,600 people). Over 35 years, this would amount to 17,668 cases of EOFTD, a rate of 0.00034 for the total population of England and Wales (57,885,400 people) and mean that 1 in 3,276 people will develop EOFTD in their lifetime.

# Summary and cross-check with national mortality statistics

The Office for National Statistics reports 706 deaths between the ages 30 to 69 years, which are attributed to AD, and 153 deaths between the ages 30 to 69 years, which are attributed to FTD. We chose the age range 30 to 69 years in order to exclude deaths most likely not caused by early-onset dementia; given the average disease duration of late-onset Alzheimer’s disease (LOAD) of 7 years(4), we excluded deaths over the age of 70. That said, deaths attributed to AD increase exponentially in the age-range 65-69 compared to younger ages, suggesting that some of them may be due to either rapidly-progressive LOAD or other causes expediting death in the presence of LOAD. This would explain the higher than expected number of reported deaths due to AD between the ages 30 to 69 years.

While deaths attributed to AD are slightly higher than expected, deaths attributed to FTD between the ages 30 to 69 years are slightly fewer than expected. Overall, these statistics are consistent with the prevalence estimates calculated earlier.

###

# Penetrance of genetic variants linked to early-onset Alzheimer’s disease and early-onset fronto-temporal dementia

In their 2016 paper, Minikel *et al.*(6) explain that, considering the low incidence and prevalence of prion disease, the number of variants that are reported pathogenic appears very high; the authors subsequently demonstrate that some reportedly pathogenic variants should be considered likely benign or incompletely penetrant based on their frequency in online population databases. Their methodology calculates the maximum expected cases in a population that is neither enriched nor depleted for the variants in question, multiplying the incidence, the proportion of genetic cases, the life expectancy of genetic cases and the number of individuals in the given population. We assumed a life expectancy of 80 years for genetic cases, a proportion of autosomal dominant genetic cases of 10% for EOAD(7) and 20% for EOFTD(8,9) and used the 141,352 individuals from the *gnomAD* online database(10). We found that the number of variants in the literature reported as at least potentially pathogenic(11) vastly exceeds what is compatible with the prevalence and proportion of genetic cases in these diseases(7), if all these reportedly deleterious and “unclear” variants were pathogenic and fully penetrant.

On the Molgen database and on the mutation database of Alzforum (Alzgene), 302 variants in *APP*, *PSEN1* and *PSEN2* were listed as deleterious and a further 21 variants were reported as having unclear pathogenicity based on case reports. In *GRN*, *MAPT* and *VCP*, 153 variants were listed as deleterious and a further 64 variants were reported as having unclear pathogenicity based on case reports. Notably, while many of the reportedly deleterious variants are not observed at all or at very low frequencies, a small number of variants were detected repeatedly, calling into question the extent of their pathogenicity and penetrance. This discrepancy highlights the importance of checking population frequency of any variant intimated to be disease causing and to assess how likely it is to have a deleterious effect.

# Published reportedly pathogenic variants

Based on their frequency in our dataset and on *gnomAD*, a number of published reportedly pathogenic variants in *APP*, *PSEN1* and *PSEN2* seem to be incompatible with being highly penetrant pathogenic mutations (Table 1S). However, as was to be expected, not all published reportedly pathogenic variants in *APP*, *PSEN1*, *PSEN2*, *GRN*, *MAPT* and *VCP*, were found in the present dataset. Indeed, of the reportedly pathogenic variants which are also present in the *gnomAD* dataset, only *PSEN1* Arg269His, *PSEN1* Pro264Leu, *GRN* Arg493Ter, *GRN* Leu469Phe, *GRN* Thr251Ser and *GRN* Glu287Asp were observed in the present case series. To permit an assessment of the other reportedly pathogenic variants, we envisaged a scenario where they would have been observed just once in our dataset and performed calculations on this basis. More specifically, *APP* Ala713Thr, *PSEN1* Ser170Phe, *PSEN1* Arg352Cys, *PSEN1* Arg358Gln, *PSEN1* Ser365Ala, *PSEN1* Val412Ile, *PSEN2* Thr430Met, *PSEN2* Ala237Val, and *PSEN2* Val214Leu are not enriched in our EOAD cohort, despite having been reported as Mendelian pathogenic variants. Penetrance for these variants was calculated to be 2% or less, with upper limits of the 95% confidence interval of less than 20%, frequently less than 10%. Indeed, penetrance for these variants is comparable to *PSEN1* Asn32Asn and *PSEN1* Asp40del, which are reported as uncertain because they were reported in only one patient each.

In the genes *GRN*, *MAPT* and *VCP* associated with FTD, most reportedly pathogenic variants appear to be very rare in the population (Table 2S); indeed *MAPT* Arg5His is the only variant listed as pathogenic with an estimated penetrance of less than 2% (95% CI: 0.3%, 9.3%). However, a large number of variants in both *GRN* and *MAPT*, which are relatively frequent in the population have been reported as associated with FTD; however, for these, the level of evidence is classified as unclear because of the number or quality of the reports. Analysis of these variants and their frequency in the population (*GRN* Asp33Glu, *GRN* Pro34Pro, *GRN* Gly35Arg, *GRN* Gly70Ser, *GRN* Val77Ile, *GRN* Arg110Gln, *GRN* Thr138Thr, *GRN* Cys139Arg, *GRN* Arg212Trp, *GRN* Pro233Gln, *GRN* Glu287Asp*, GRN* Arg298His, *GRN* Pro392Pro, *GRN* Arg432Cys, *GRN* His447His, *GRN* Cys495Cys, *GRN* Val514Met, *GRN* Val519Met, *GRN* Cys521Tyr; *MAPT* Ala41Thr, *MAPT* Gly86Ser, *MAPT* Ala297Val, *MAPT* Ser318Leu, *MAPT* Ser427Phe, *MAPT* Arg448Ter) revealed that they are unlikely to be highly penetrant with a calculated penetrance of less than 3% and an upper limit of the confidence interval of less than 20% penetrance.

# Variants detected in the present dataset

Following the method set out by Minikel *et al.*(6), all variants in *APP*, *PSEN1*, *PSEN2*, *GRN*, *MAPT* and *VCP* that were observed both in our dataset and on *gnomAD* were assessed for their likely penetrance. For calculation purposed, the EOAD and the EOFTD cohorts included 768 and 447 cases, respectively. We limited our analysis to these subsets because it was not possible to reliably estimate the prevalence of genetically-determined dementia in old age. As set out above, the baseline lifetime risk for EOAD and EOFTD was assumed to be 0.031% and 0.034%, respectively, and compared against population data from the *gnomAD* database, which contains information from 141,352 individuals. These variants are listed in Table 3S.

# Figure 1S: Age at clinical onset (AAO) in patients with a DV, per gene

Box plots represent the interquartile range of age at onset (AAO) in each gene category with the median marked by a line; the whiskers represent the minimum and maximum AAO identified in each gene, except for values more than 1.5x the interquartile range from the median which are represented as outliers. Values between 1.5x and 3x the interquartile range a represented as points, while extreme values more than 3x the interquartile range from the median are represented as an asterix. In addition to the 294 cases from this dataset for whom age of onset was available, this graph contains data from an additional 54 patients with DVs who had been previously tested clinically at the MRC Prion Unit. These were added to increase sample size and more reliably report the range of AAO observed for DVs in each gene in the UK. We note that AAO for PSEN1 appears to be substantially earlier in the literature(12,13) which may relate to different methods of ascertainment of patients with a DV.

# Table 1S: Reported pathogenic variants in Alzheimer’s Disease genes *APP*, *PSEN1* and *PSEN2*

Listed are reportedly pathogenic variants, which were also found on *gnomAD*, with their respective frequency in the population and likely penetrance. *PSEN1* Arg269His, *PSEN1* Pro264Leu and *APP* Ala713Thr were identified in our tested sample dataset; all three are reportedly pathogenic. While *PSEN1* Arg269His and *PSEN1* Pro264Leu are compatible with fully penetrant pathogenic mutations, *APP* Ala713Thr is likely to have a very limited penetrance based on its frequency in the population. For all other variants, penetrance calculations were based on a hypothetical count of one in our sample (ie calculations are biased towards higher penetrance). 322 variants in *APP*, *PSEN1* and *PSEN2* were listed as pathogenic on the Molgen AD/FTD database(11) and/or Alzforum; variants listed as “pathogenic nature unclear” on Molgen were also included and marked as such in the column “Reported”. The allele count and frequency of the protein changes induced by these variants on *gnomAD* is also included, except for copy number variants (CNVs).

| **Gene** | **Mutation** | **Exon** | **Domain** | **gnomAD Allele Count** | **gnomAD Allele Freq.** | **Source** | **Reported** | **Penetrance (95% CI)** |
| --- | --- | --- | --- | --- | --- | --- | --- | --- |
| ***Most likely reduced penetrance*** | | | | |  |  |  |  |
| *PSEN1* | Val94Met | EX4 | TM-I | 2 | 0.0008% | molgen | deleterious | 5.2 (0.4%, 64.4%) |
| *PSEN1* | Thr354Ile | EX10 | HL-VI b | 2 | 0.0008% | molgen | unclear | 5.2 (0.4%, 64.4%) |
| *PSEN2* | Gln228Leu | EX7 | TM-V | 2 | 0.0008% | molgen | deleterious | 5.2 (0.4%, 64.4%) |
| *PSEN1* | Arg108Gln | EX4 | HL-I | 2 | 0.0008% | molgen | deleterious | 5.2 (0.4%, 64.3%) |
| *PSEN2* | Val148Ile | EX5 | TM-II | 2 | 0.0008% | molgen | deleterious | 5.2 (0.4%, 64%) |
| *PSEN1* | Glu123Lys | EX5 | HL-I | 3 | 0.0011% | molgen | deleterious | 3.9 (0.4%, 41.3%) |
| *PSEN2* | Lys161Arg | EX5 |  | 3 | 0.0011% | alzgene | deleterious | 3.8 (0.4%, 40.2%) |
| *PSEN1* | Gly206Ala | EX7 | TM-IV | 3 | 0.0012% | molgen | deleterious | 3.4 (0.3%, 34.8%) |
| *PSEN2* | Leu238Pro | EX7 | TM-V | 3 | 0.0012% | molgen | deleterious | 3.4 (0.3%, 34.8%) |
| *PSEN1* | Ala79Val | EX4 | N-Term | 4 | 0.0014% | molgen | deleterious | 2.9 (0.3%, 26.9%) |
| *PSEN1* | Arg352dup | EX10 | HL-VI b | 4 | 0.0014% | molgen | unclear | 2.9 (0.3%, 26.9%) |
| *PSEN1* | Ser365Ala | EX10 | HL-VI b | 5 | 0.0020% | molgen | deleterious | 2.1 (0.3%, 16.8%) |
| *PSEN1* | Ser170Phe | EX6 | TM-III | 8 | 0.0028% | molgen | deleterious | 1.4 (0.2%, 10.4%) |
|  |  |  |  |  |  |  |  |  |
| **Most likely fully penetrant (95%CI >100% penetrance)** | | | | |  |  |  |  |
| *PSEN1* | Arg269His | EX8 | HL-VI a | 1 | 0.0004% | molgen | deleterious |  |
| *PSEN1* | Pro264Leu | EX8 | HL-VI a | 1 | 0.0004% | molgen | deleterious |  |
| *PSEN1* | Ile202Phe | EX7 | TM-IV | 1 | 0.0004% | molgen | deleterious |  |
| *PSEN1* | His214Tyr | EX7 | HL-IV | 1 | 0.0004% | molgen | deleterious |  |
| *PSEN1* | Leu219Phe | EX7 | HL-IV | 1 | 0.0004% | molgen | deleterious |  |
| *PSEN1* | Ile408Thr | EX11 | TM-VIII | 1 | 0.0004% | molgen | deleterious |  |
| *PSEN2* | Ala85Val | EX4 | N-Term | 1 | 0.0004% | molgen | deleterious |  |
|  |  |  |  |  |  |  |  |  |
| **Most likely benign or only small increase in risk (95%CI <10% penetrance)** | | | | | |  |  |  |
| *APP* | Ala713Thr | EX17 | TM-I | 26 | 0.0092% | molgen | deleterious |  |
| *PSEN1* | Val412Ile | EX11 | TM-VIII | 1 | 0.0033% | molgen | deleterious |  |
| *PSEN2* | Thr430Met | EX12 |  | 9 | 0.0036% | alzgene | deleterious |  |
| *PSEN1* | Arg352Cys | EX10 | HL-VI b | 12 | 0.0043% | molgen | deleterious |  |
| *PSEN1* | Arg358Gln | EX10 | HL-VI b | 11 | 0.0044% | molgen | deleterious |  |
| *PSEN2* | Ala237Val | EX7 | TM-V | 14 | 0.0056% | molgen | deleterious |  |
| *PSEN2* | Val214Leu | EX7 |  | 65 | 0.0230% | alzgene | deleterious |  |
| *PSEN1* | Asn32Asn | EX4 | N-Term | 12 | 0.0043% | molgen | unclear |  |
| *PSEN1* | Asp40del | EX4 | N-Term | 39 | 0.0138% | molgen | unclear |  |

#

# Table 2S: Reportedly pathogenic variants in Frontotemporal Dementia genes *GRN*, *MAPT* and *VCP*

Listed are reportedly pathogenic variants, which were also found on *gnomAD*, with their respective frequency in the population and likely penetrance, as well as reportedly unclear variants with an upper penetrance 95%CI >50%. *GRN* Arg493Ter, *GRN* Arg110Ter, *GRN* Leu469Phe and *GRN* Thr251Ser were identified in our dataset; the first two are reportedly pathogenic. *GRN* Arg493Ter, *GRN* Arg110Ter, *GRN* Leu469Phe and *GRN* Thr251Ser are all compatible with being fully penetrant pathogenic mutations based on their frequency in cases and in the population. For all other variants, penetrance calculations were based on a hypothetical count of one in our dataset. 217 variants in *GRN*, *MAPT* and *VCP* were listed as pathogenic on the Molgen AD/FTD database and/or Alzforum; variants listed as “unclear pathogenicity” and reported in cases were also included and marked as such in the column “Reported”. The allele count and frequency of the protein changes induced by these variants on *gnomAD* is also included, except for copy number variants (CNVs).

| **Gene** | **Mutation** | **Exon** | **Domain** | **GnomAD Allele Count** | **GnomAD Allele Freq.** | **Source** | **Reported** | **Penetrance (95% CI)** |
| --- | --- | --- | --- | --- | --- | --- | --- | --- |
| *MAPT* | Gly55Arg | Ex2 |  | 3 | 0.001% | alzforum | deleterious | 7.6% (0.7%, 81.3%) |
| *VCP* | Arg191Gln | EX5 | Linker 1 | 4 | 0.002% | molgen | deleterious | 5.1% (0.6%, 45.5%) |
| *GRN* | Cys222Tyr | EX7 | GranB | 3 | 0.001% | molgen | unclear | 6.8% (0.7%, 68.5%) |
| *GRN* | Arg535Ter | EX12 | GranE | 3 | 0.001% | molgen | unclear | 6.8% (0.7%, 68.5%) |
| *MAPT* | Arg5Cys | EX1 |  | 3 | 0.001% | alzforum | unclear | 6.8% (0.7%, 68.5%) |
| *GRN* | Arg564Cys | EX13 | GranE | 3 | 0.001% | molgen | unclear | 6.7% (0.7%, 67.7%) |
| *VCP* | Arg95His | EX3 | CDC48 | 4 | 0.001% | molgen | unclear | 5.7% (0.6%, 53%) |
|  |  |  |  |  |  |  |  |  |
| **Most likely fully penetrant (95%CI >100% penetrance)** | | | | |  |  |  |  |
| *GRN* | Arg493Ter | EX12 | GranD | 1 | 0.000% | molgen | deleterious |  |
| *GRN* | Arg110Ter | EX4 | GranG | 1 | 0.000% | molgen | deleterious |  |
| *GRN* | Leu469Phe | EX11 | GranD | 1 | 0.000% | molgen | unclear |  |
| *GRN* | Thr251Ser | EX8 | GranB | 3 | 0.001% | molgen | unclear |  |
| *GRN* | Pro127fs | EX5 | GranF | 1 | 0.000% | molgen | deleterious |  |
| *VCP* | Arg159Cys | EX5 | CDC48 | 1 | 0.000% | molgen | deleterious |  |
| *GRN* | Cys253Ter | EX8 | GranB | 1 | 0.000% | molgen | deleterious |  |
| *GRN* | Thr382fs | EX10 | GranC | 1 | 0.000% | molgen | deleterious |  |
| *GRN* | Thr382fs | EX10 | GranC | 1 | 0.000% | molgen | deleterious |  |
| *GRN* | Gln130fs | EX5 | GranF | 2 | 0.001% | molgen | deleterious |  |
| *GRN* | Gln130fs | EX5 | GranF | 2 | 0.001% | molgen | deleterious |  |
| *VCP* | Arg159His | EX5 | CDC48 | 2 | 0.001% | molgen | deleterious |  |
| *VCP* | Arg95Cys | EX3 | CDC48 | 2 | 0.001% | molgen | deleterious |  |
| *GRN* | Cys105Arg | EX4 | GranG | 1 | 0.000% | molgen | unclear |  |
| *GRN* | Arg547Cys | EX12 | GranE | 1 | 0.000% | molgen | unclear |  |
| *MAPT* | Val75Ala | EX3 | - | 1 | 0.000% | molgen | unclear |  |
| *GRN* | Ala276Val | EX8 | InterBA | 2 | 0.001% | molgen | unclear |  |
| *GRN* | Pro451Leu | EX11 | GranD | 2 | 0.001% | molgen | unclear |  |
|  |  |  |  |  |  |  |  |  |
| **Most likely benign or only small increase in risk (95%CI <10% penetrance)** | | | | | |  |  |  |
| *MAPT* | Arg5His | EX1 | N-Term | 14 | 0.01% | molgen | deleterious |  |

# Table 3S: Penetrance of variants observed in our own dataset in *APP, PSEN1, PSEN2, GRN, MAPT* and *VCP*

The EOAD and the EOFTD cohorts included 757 and 421 cases, respectively, and the baseline lifetime risk for EOAD and EOFTD was assumed to be 0.031% and 0.034%, respectively. Variants were compared against population data from the *gnomAD* database, which contains information from 141,352 individuals.

| **Gene** | **Variant** | **Classification** | **Cohort** | **Case Allele Count** | **Case Allele Freq.** | **gnomAD Allele Count** | **gnomAD Allele Freq.** | **Penetrance and CI** |
| --- | --- | --- | --- | --- | --- | --- | --- | --- |
| *MAPT* | Gly389Arg | Deleterious | EOFTD | 2 | 0.0048 | 4 | 0.000016 | 10.16% (1.6, 63.5%) |
| *PSEN1* | Ile227Val | Likely Deleterious | EOAD | 1 | 0.0013 | 4 | 0.000014 | 2.88% (0.3, 26.9%) |
| *GRN* | Thr251Ser | Possible | EOAD + EOFTD | 2 | 0.0016 | 3 | 0.000011 | 10.09% (1.3, 76.2%) |
| *PSEN2* | Tyr195Cys | Possible | EOFTD | 1 | 0.0024 | 4 | 0.000016 | 5.08% (0.6, 45.1%) |
| *PSEN1* | Arg42Leu | Possible | EOAD | 2 | 0.0026 | 5 | 0.00002 | 4.14% (0.7, 23.7%) |
| *MAPT* | Gly415Ser | Possible | EOFTD | 1 | 0.0024 | 6 | 0.000021 | 3.81% (0.5, 30.2%) |
| *PSEN2* | Leu135Arg | Possible | EOFTD | 1 | 0.0024 | 1 | 0.000033 | 2.44% (0.4, 16.6%) |
| *GRN* | Thr268Met | Possible | EOAD | 1 | 0.0013 | 5 | 0.000018 | 2.31% (0.3, 19.7%) |
| *FUS* | Gly225Ser | Possible | EOAD | 1 | 0.0013 | 5 | 0.000019 | 2.12% (0.3, 17.5%) |
| *GRN* | Pro458Leu | Possible | EOFTD | 1 | 0.0024 | 12 | 0.000043 | 1.89% (0.3, 12%) |
| *PSEN2* | Leu225Pro | Risk factor | EOAD | 1 | 0.0013 | 2 | 0.000008 | 5.17% (0.4, 64.3%) |
| *MAPT* | c.*16G>A | Uncertain | EOAD | 1 | 0.0013 | 2 | 0.000008 | 5.15% (0.4, 64%) |
| *MAPT* | Asn167Ser | Uncertain | EOFTD | 1 | 0.0024 | 9 | 0.000032 | 2.54% (0.4, 17.5%) |
| *APP* | Gly657Arg | Uncertain | EOFTD | 1 | 0.0024 | 1 | 0.000033 | 2.44% (0.4, 16.6%) |
| *APP* | Arg16Gln | Uncertain | EOAD | 1 | 0.0013 | 5 | 0.00003 | 1.36% (0.2, 9.5%) |
| *MAPT* | Gly201Ser | Uncertain | EOAD | 1 | 0.0013 | 9 | 0.000033 | 1.25% (0.2, 8.6%) |
|  |  |  |  |  |  |  |  |  |
| **Most likely fully penetrant (95%CI >100% penetrance)** | | | | |  |  |  |  |
| *GRN* | Arg493Ter | Deleterious | EOFTD | 4 | 0.0095 | 1 | 0.000004 |  |
| *PSEN1* | Arg269His | Deleterious | EOAD | 3 | 0.004 | 1 | 0.000004 |  |
| *MAPT* | Arg406Trp | Deleterious | EOFTD | 6 | 0.0143 | 4 | 0.000016 |  |
| *MAPT* | Pro301Leu | Deleterious | EOAD + EOFTD | 2 | 0.0017 | 1 | 0.000005 |  |
| *GRN* | Arg110Ter | Deleterious | EOFTD | 1 | 0.0024 | 1 | 0.000004 |  |
| *MAPT* | Lys257Thr | Deleterious | EOFTD | 1 | 0.0024 | 1 | 0.000004 |  |
| *PSEN1* | Pro264Leu | Deleterious | EOAD | 1 | 0.0013 | 1 | 0.000004 |  |
| *PSEN1* | Ser132Ala | Likely Deleterious | EOAD + EOFTD | 2 | 0.0017 | 1 | 0.000004 |  |
| *PSEN1* | Gln15His | Possible | EOFTD | 1 | 0.0024 | 1 | 0.000004 |  |
| *PSEN1* | Pro303Leu | Possible | EOFTD | 1 | 0.0024 | 1 | 0.000004 |  |
| *PSEN2* | Arg62Cys | Possible | EOFTD | 1 | 0.0024 | 1 | 0.000004 |  |
| *GRN* | Leu469Phe | Possible | EOFTD | 1 | 0.0024 | 1 | 0.000004 |  |
| *PSEN1* | Asn39Tyr | Possible | EOAD | 1 | 0.0013 | 1 | 0.000004 |  |
| *MAPT* | Arg194His | Possible | EOFTD | 1 | 0.0024 | 2 | 0.000008 |  |
| *PSEN2* | Arg163Cys | Risk factor | EOFTD | 2 | 0.0048 | 1 | 0.000004 |  |
| *VCP* | Val133Ile | Uncertain | EOFTD | 1 | 0.0024 | 1 | 0.000004 |  |
| *FUS* | Pro459Leu | Uncertain | EOAD | 1 | 0.0013 | 1 | 0.000004 |  |
| *MAPT* | c.*19C>A | Uncertain | EOFTD | 1 | 0.0024 | 2 | 0.000008 |  |
|  |  |  |  |  |  |  |  |  |
| **Most likely benign or only small increase in risk (95%CI <10% penetrance)** | | | | | |  |  |  |
| *APP* | Ala713Thr | Likely deleterious | EOAD | 1 | 0.0013 | 26 | 0.000092 |  |
| *GRN* | Glu287Asp | Possible | EOAD | 1 | 0.0013 | 10 | 0.000035 |  |
| *GRN* | Ala582Thr | Possible | EOFTD | 1 | 0.0024 | 21 | 0.000075 |  |
| *GRN* | Arg110Gln | Possible | EOFTD | 1 | 0.0024 | 22 | 0.000087 |  |
| *GRN* | Val514Met | Possible | EOAD | 1 | 0.0013 | 13 | 0.000046 |  |
| *FUS* | Pro18Ser | Possible | EOFTD | 1 | 0.0024 | 33 | 0.000117 |  |
| *GRN* | Arg535Gln | Possible | EOAD | 1 | 0.0013 | 21 | 0.000083 |  |
| *FUS* | Pro431Leu | Possible | EOAD | 1 | 0.0013 | 31 | 0.000111 |  |
| *GRN* | Asn39Tyr | Possible | EOAD | 1 | 0.0013 | 47 | 0.000168 |  |
| *GRN* | His340Leu | Possible | EOAD | 1 | 0.0013 | 24 | 0.000182 |  |
| *GRN* | c.-56T>G | Possible | EOAD | 1 | 0.0013 | 24 | 0.000793 |  |
| *PSEN2* | Ser130Leu | Risk factor | EOAD + EOFTD | 6 | 0.0051 | 177 | 0.000627 |  |
| *VCP* | Ile27Val | Risk factor | EOAD + EOFTD | 4 | 0.0034 | 183 | 0.000647 |  |
| *MAPT* | Ala152Thr | Risk factor | EOAD + EOFTD | 8 | 0.0068 | 403 | 0.00143 |  |
| *PSEN2* | Met174Val | Risk factor | EOAD | 4 | 0.0053 | 166 | 0.000587 |  |
| *MAPT* | Ala152Thr | Risk factor | EOAD + EOFTD | 2 | 0.0017 | 403 | 0.00143 |  |
| *PSEN2* | Arg62His | Risk factor | EOAD + EOFTD | 7 | 0.0059 | 2642 | 0.00936 |  |
| *APP* | Arg16Gln | Uncertain | EOAD | 1 | 0.0013 | 5 | 0.00003 |  |
| *MAPT* | Gly201Ser | Uncertain | EOAD | 1 | 0.0013 | 9 | 0.000033 |  |

Table 4S: Tabulation of novel DVs**.** Comments refer to the main lines of evidence used to justify the classification based on rules defined by Tables 1a and b.

| **Pathogenicity** | **Times observed** | **AAO** | **Type** | **Gene** | **Alleles / gnomAD** | **HGVSProtein (or coding if no change in protein)** | **Comment** |
| --- | --- | --- | --- | --- | --- | --- | --- |
| **Likely Deleterious** | 3 | 65.5, 55.5, 55.6 | missense | CSF1R | 15 | p.Leu868Arg | no variant at this position on exac. Leu 868Pro reported pathogenic |
| **Deleterious** | 3 | 64, 62 | frame-shift | GRN | 0 | p.Gln130Serfs | frameshift mutations in GRN cause haploinsufficiency and disease. Several mutations at this codon causing deleterious frameshift mutations have been reported on molgen |
| **Deleterious** | 3 | Unknown, 25.5, 64 | frame-shift | GRN | 0 | p.Ser78Phefs | not on exac, causes a premature stop codon. frameshift mutations in GRN reported pathogenic, pathogenic frameshift mutation on molgen in the adjacent codon |
| **Deleterious** | 2 | Unknown, Unknown | frame-shift | GRN | 0 | p.Ser129Lysfs | frameshift mutation in GRN, adjacent to two other frameshift mutation at codon 130 reported deleterious |
| **Likely Deleterious** | 2 | 52, 52, | missense | MAPT | 0 | p.Gln351Arg | not on molgen, not on exac, not on exome variant server. In silico predictions conflicting but mostly damaging. Aminoacid change form uncharged polarised to positively charged, only slightly bigger. |
| **Likely Deleterious** | 2 | 50, 59 | missense | MAPT | 0 | p.Gly271Arg | not on molgen, not on exac, not on EVS, not on google. Big aminoacid change between a synonymous (benign) and a pathogenic mutation |
| **Likely Deleterious** | 1 | 44 | missense | CSF1R | 0 | p.Ala891Pro | not on exac, not on evs. In silico not consistent. Mutation located in intracellular tyrosine kinase domain Clinical description fits Hereditary Diffuse Leukoencephalopathy with spheroids. Big amino acid change from small hydrophobic alanine to big special case proline |
| **Deleterious** | 1 | 39.87123 | frame-shift | CSF1R | 0 | p.Asp829Valfs | Not on gnomad. Adjacent to a deleterious mutation and many more in the immediate vicinity in the tyrosine kinase domain where most deleterious mutations are located. Big amino acid change with charge change from positively charged to hydrophobic in the catalytic intracellular domain with an additional frameshift. On other chromosome from Glu694Lys variant in same patient, could be additive / recessive. |
| **Likely Deleterious** | 1 | 41 | missense | CSF1R | 0 | p.His776Tyr | Not previously reported, predicted damaging in silico but not strongly, not in exac. In mutation hotspot according to Guerreiro et al. 2013, adjacent mutations either similar or less pronounced than this big aminoacid change going from charged to hydrophobic |
| **Deleterious** | 1 | - | frame-shift | GRN | 0 | c.522_523insTGTGAAGACAGGGTGCACTGCTGTC | Not an artefact. Frameshift mutation relatively early in the gene. Loss of function and haploinsufficiency known disease mechanism in GRN |
| **Deleterious** | 1 | 55.61096 | frame-shift | GRN | 0 | p.Asp254Valfs | This result confirms the diagnosis of a GRN-related dementia. This 2-bp duplication in granulin exon 8 causes a frameshift and a premature STOP, 3 codons downstream. Although this specific sequence change has not been previously reported, several pathogenic frameshift mutations in GRN have been previously described in the literature1. |
| **Likely Deleterious** | 1 | 77.5 | intronic | GRN | 0 | p.c.264+1G>A | mutation in a splice site, after input of exon , Human Splice Finder predict broken WT site and Alteration of the WT donor site, most probably affecting splicing. |
| **Deleterious** | 1 | 58.89315 | frame-shift | GRN | 0 | p.Cys260Valfs | This result confirms the diagnosis of a GRN-related dementia. This 1-bp duplication in granulin exon 8 causes a frameshift and a premature stop 14 codons downstream. Although this specific sequence change has not been previously reported, several pathogenic frameshift mutations in GRN have been previously described in the literature1. |
| **Deleterious** | 1 | 67.14521 | frame-shift | GRN | 0 | p.Cys482Ter | This result confirms the diagnosis of a GRN-related dementia. This substitution in granulin exon 12 causes a premature STOP codon. Although this specific sequence change has not been previously reported, several pathogenic nonsense and frameshift mutations in GRN causing similar protein effects have been described in the literature1. |
| **Deleterious** | 1 | 60 | frame-shift | GRN | 0 | p.Leu187Argfs | This result supports the diagnosis of a GRN-related dementia. This 1-bp deletion in granulin exon 6 causes a frameshift and a premature stop 69 codons downstream. Although this specific sequence change has not been previously reported, several pathogenic frameshift mutations in GRN have been previously described in the literature |
| **Deleterious** | 1 | 72.4274 | frame-shift | GRN | 0 | p.Met1 | This result is consistent with a diagnosis of GRN-related dementia. The c.1179G>A p.? variant has not been previously reported in the literature, though it is likely to be pathogenic. It occurs at a highly conserved splice site at an intron-exon boundary and is therefore predicted to affect GRN splicing. Analysis of affected family members would assist in the interpretation of this result. |
| **Likely Deleterious** | 1 | 59 | in-frame | GRN | 0 | p.Ser449_Thr455del | not on exac, not on google. Deletion slightly earlier in the gene has been described to cause young onset neurodegeneration, but insufficient information about exon 11. CADD phred 22.8 |
| **Deleterious** | 1 | - | missense | MAPT | 0 | p.Gly303Ser | Gly303Val is called path on Molgen, 1 family. Described in a pedigree with segregation |
| **Likely Deleterious** | 1 | 58.5 | missense | MAPT | 0 | p.His362Tyr | not on exac, not on google. In silico prediction disagree, but big amino acid change with big charge change and next to a pathogenic mutation with a smaller aminoacid change without charge change |
| **Deleterious** | 1 | 56.52877 | missense | NOTCH3 | 0 | p.Arg640Cys | This result is consistent with a diagnosis of Cerebral Autosomal Dominant Arteriopathy with Subcortical Infarcts and Leukoencephalopathy (CADASIL). The c.1918C>T p. (Arg640Cys) variant has not been previously reported in the literature, though it is likely to be pathogenic. In keeping with many disease-causing mutations in NOTCH3, this substitution involves a change to a Cysteine amino acid within an EGF-like domain of the NOTCH3 protein. This variant has been reported to the Leiden Open Variation Database (LOVD) 1, having been detected in a patient with CADASIL. It is also recorded in the ExAC variation database2 at a global minor allele frequency of 0.0025%. In order to confirm a diagnosis of CADASIL, review of brain MRI for the characteristic white matter intensities is recommended. |
| **Likely Deleterious** | 1 | 47 | missense | PSEN1 | 0 | p.Ala137Thr | not on molgen, but in mutation hotspot. Not on exac, not on exome variant server. In silico predictions conflicting |
| **Likely Deleterious** | 1 | 57.5 | intronic | PSEN1 | 0 | c.869-1G>A | CADD score 26.7, not on exac, intronic mutation. Human Splice Finder predicts a broken WT site and Alteration of the WT acceptor site, most probably affecting splicing. |
| **Likely Deleterious** | 1 | 49 | missense | PSEN1 | 0 | p.Gln222Pro | not on exac. not on molgen, but 2 mutations at same locus pathogenic (one change to Arg, one to His); these are both charged aminoacid in a transmembrane domain and Proline is a special case, but with a very different sidechain from Gln |
| **Likely Deleterious** | 1 | 38.5 | in-frame | PSEN1 | 0 | p.Leu171Tyr | not on exac. Similar mutation (L166H) reported pathogenic at same location, this variant has no charge change but a change in bulkiness. |
| **Likely Deleterious** | 1 | 43.5 | missense | PSEN1 | 0 | p.Pro433Ser | not on exac. predicted deleterious. on the very edge of the intermembrane domain, next to several deleterious mutations, but only to one side, quite significant aminoacid change to a much smaller one |
| **Likely Deleterious** | 1 | 55 | missense | PSEN1 | 0 | p.Thr122Ala | not on exac. aminoacid change from polar to hydrophobic in the 1st luminal part of PSEN1, close and adjacent to pathogenic mutations with no charge change |
| **Deleterious** | 1 | 54.50685 | missense | PSEN1 | 0 | p.Val142Ile | This result is consistent with a diagnosis of Alzheimer's disease in this patient. The PSEN1 c.424G>A p. (Val142Ile) variant has not been previously reported in the literature or to public databases of genetic variation (ExAC, EVS, 1000G), though it is likely to be pathogenic. It causes a missense change at a highly conserved amino acid located within the second transmembrane domain of the protein, a region in which several pathogenic missense mutations have been previously reported. Analysis of affected family members would assist in the interpretation of this result. |
| **Likely Deleterious** | 1 | 64.5 | missense | PSEN1 | 0 | p.Val393Phe | not on exac, big aminoacid change in size, deleterious mutations in both adjacent codons (with smaller aminoacid changes), predicted deleterious in silico, CADD phredd 35 |
| **Likely Deleterious** | 1 | 30 | missense | PSEN2 | 0 | p.Val150Met | Not in Exac, known pathogenic mutation at 148 v148i (similar aminoacid), reported in young onset Alzheimer patient with a family history in a thesis |
| **Likely Deleterious** | 1 | 65 | missense | VCP | 0 | p.Pro137Ser | Mutation at same location has been described in various pedigrees related to dementia with Paget’s disease or with myopathy. The amino acid change caused by this variant compared to the published one is slightly less dramatic, but still very similar with a change from proline to an uncharged polar side chain instead of a hydrophobic one |

# Table 5Sa: Evidence used to classify variants according to their pathogenicity level

Variants identified in a sample were classified according to the information available about them. This included the type of mutation in question, its position in the gene and/or protein, its frequency in online population databases, *in silico* predictions of effects on proteins, and whether it had previously been reported in families, single cases or controls.

| Evidence level | Criteria |
| --- | --- |
| Pathogenic  Strong | 1) Coding amino-acid change previously published as deleterious with evidence of segregation in more than one pedigree or in multiple unrelated patients with the same phenotype  2) Null variant in a gene where loss of function (LOF) is a known disease mechanism (caveat LOF variants at extreme 3' end)  3) Variant in a gene associated with an expected very rare pathology (e.g. *PRNP* mutation and prion pathology)  4) Explained mechanism of pathophysiology of variant using *in vitro* or *in vivo* studies  5) Found in a mutational hotspot i.e. a domain where many other pathogenic mutations are seen, generally with additionally support from *in silico* prediction software |
| Pathogenic Moderate | 1) Coding amino-acid change previously and justifiably published as deleterious but without evidence of segregation or in a single pedigree/patient  2) Novel missense change at an amino acid residue where a different pathogenic missense change has been seen  3) A very different amino-acid change at the same site or next to one with a less dramatic amino-acid change but deleterious  4) In a gene the mechanism of which is understood and the effect of the variant is in keeping with that mechanism;  5) Protein length changes as a result of in-frame deletions/insertions in a nonrepeat region or stop-loss variants  6) Mutation in a gene associated with a rare pathology in a case with a compatible clinical syndrome  7) Intronic variant affecting splicing or protein length |
| Pathogenic Supporting | 1) Variant with a major amino-acid change near or in a functional domain (e.g. active site of an enzyme) but not in a mutational hotspot  2) Multiple lines of computational evidence support a deleterious effect on the gene or gene product (conservation, evolutionary, splicing impact, etc.) Caveat: Because many in silico algorithms use the same or very similar input for their predictions, each algorithm should not be counted as an independent criterion  3) Reported in both cases and controls, but more cases than controls (statistically significant in a study) |
| Pathogenic  Risk factor | 1) Previously reported as risk factor, either variant itself or clear established pattern in gene  2) >1 in 10000 in *gnomAD*;  3) The prevalence of the variant in affected individuals is significantly increased compared with the prevalence in controls |
| Benign Independent | Allele frequency >5% on *gnomAD*, or 1000 genomes project |
| Benign  Strong | 1) Allele frequency >1% on *gnomAD*;  2) Reported benign in multiple pedigrees or with insight into gene/protein mechanism  3) Allele frequency is greater than expected for disorder  4) Lack of segregation in affected members of a family, caveat: phenocopies and penetrance  5) Seen in equal or greater frequencies in controls than cases |
| Benign  Moderate | 1) Allele frequency over 0.1% on *gnomAD*  2) Reported benign in one case or pedigree  3) Genetic mechanism inconsistent with pathological phenotype, or known mutation spectrum |
| Benign  Supporting | 1) Missense variant in a gene for which primarily truncating variants are known to cause disease or the mechanism is very specific and known  2) Multiple lines of computational evidence suggest no impact on gene or gene product (conservation, evolutionary, splicing impact, etc.)  3) A synonymous (silent) variant for which splicing prediction algorithms predict no impact to the splice consensus sequence |

Table 5Sb: Criteria for variant classification. The evidence available about each variant was combined to determine its likely effect and likelihood of causing disease

| Pathogenicity | Algorithm |
| --- | --- |
| Deleterious | Found in patient(s) and not controls OR in significant excess in patients AND seen on *gnomAD* at less than 1 in 50,000;  AND Pathogenic Strong evidence 1) OR 2),  PLUS one additional Pathogenic Strong or two Pathogenic moderate or one Pathogenic moderate and one Pathogenic Supporting criterion |
| Likely deleterious | The prevalence of the variant in affected individuals is significantly increased compared with the prevalence in controls, or only seen on *gnomAD* at less than 1 in 10,000;  AND Pathogenic Moderate evidence 1) OR 2) OR 3)  AND one additional Pathogenic Strong or Moderate or Supporting criteria. |
| Possibly deleterious | Found on *gnomAD* at less than 1 in 5000 and at least one Supporting criterion |
| Uncertain | Insufficient or conflicting evidence  Missense mutation not nearby other missense mutations thought to be pathogenic |
| Likely benign | One Benign Strong criteria OR one Benign Moderate AND one Benign Supporting criteria OR two Benign Supporting criteria |
| Benign | Benign Independent OR one Benign Strong evidence criterion AND two further Benign Moderate or Benign Supporting criteria |
| Risk factor | Previously reported as risk factor, either variant itself or clear established pattern in gene,  AND >1 in 10000 in *gnomAD*;  AND the prevalence of the variant in affected individuals is significantly increased compared with the prevalence in controls |

# Table 6S: Number of variants in each pathogenicity class observed in the present dataset

The total number of variants in each variant pathogenicity class identified in each of the respective cohorts is shown as well as a percentage of the number of cases in each cohort for those of uncertain or at least possible pathogenicity. In 1052 AD patients, 15 novel DVs were identified; in 794 FTD patients, 24 novel DVs were identified, in prion patients one novel DV was identified, and in DemMot patients three novel DVs were identified. In 243 patient and control samples we could not be certain of age at onset, therefore the sum of Early and Late-Onset does not equal All Ages.

| **N Variants** |  | | **Deleterious** | **Likely deleterious** | **Novel DVs** | **Possible** | **Uncertain** | **Likely benign** | **Benign** | **Risk Factor** | **Synonymous** | **Total N Cohort** |
| --- | --- | --- | --- | --- | --- | --- | --- | --- | --- | --- | --- | --- |
| **Early-Onset** | **AD** | 38 (4.6%) | | 19 (2.3%) | 12 (1.5%) | 34 (4.2%) | 70 (8.6%) | 39 (4.8%) | 545 (66.6%) | 147 (18%) | 1957 (239.2%) | 818 |
|  | **FTD** | 112 (23%) | | 10 (2.1%) | 14 (2.9%) | 13 (2.7%) | 65 (13.3%) | 24 (4.9%) | 314 (64.5%) | 81 (16.6%) | 1364 (280.1%) | 487 |
|  | **Prion** | 57 (35%) | | 1 (0.6%) | 0 (0%) | 4 (2.5%) | 2 (1.2%) | 5 (3.1%) | 93 (57.1%) | 29 (17.8%) | 563 (345.4%) | 163 |
|  | **DemMot** | 19 (4.3%) | | 4 (0.9%) | 2 (0.4%) | 14 (3.1%) | 20 (4.5%) | 24 (5.4%) | 278 (62.2%) | 82 (18.3%) | 1221 (273.2%) | 447 |
|  | **Controls** | 0 (0%) | | 0 (0%) | 0 (0%) | 0 (0%) | 0 (0%) | 47 (1175%) | 5 (125%) | 2 (50%) | 17 (425%) | 4 |
|  | **Total** | 226 (11.8%) | | 34 (1.8%) | 29 (1.5%) | 65 (3.4%) | 157 (8.2%) | 139 (7.2%) | 1234 (64.3%) | 341 (17.8%) | 5122 (266.9%) | 1919 |
| **Late-Onset** | **AD** | 9 (4%) | | 4 (1.8%) | 3 (1.3%) | 12 (5.3%) | 9 (4%) | 91 (40.3%) | 166 (73.5%) | 39 (17.3%) | 698 (308.8%) | 226 |
|  | **FTD** | 16 (9.9%) | | 2 (1.2%) | 3 (1.9%) | 6 (3.7%) | 20 (12.3%) | 59 (36.4%) | 84 (51.9%) | 34 (21%) | 451 (278.4%) | 162 |
|  | **Prion** | 5 (7.5%) | | 1 (1.5%) | 0 (0%) | 2 (3%) | 1 (1.5%) | 17 (25.4%) | 29 (43.3%) | 21 (31.3%) | 204 (304.5%) | 67 |
|  | **DemMot** | 2 (1.1%) | | 1 (0.6%) | 0 (0%) | 8 (4.5%) | 6 (3.4%) | 85 (48.3%) | 100 (56.8%) | 17 (9.7%) | 471 (267.6%) | 176 |
|  | **Controls** | 2 (0.4%) | | 0 (0%) | 0 (0%) | 5 (1.1%) | 12 (2.7%) | 3 (0.7%) | 314 (70.1%) | 49 (10.9%) | 1415 (315.8%) | 448 |
|  | **Total** | 34 (3.2%) | | 8 (0.7%) | 6 (0.6%) | 33 (3.1%) | 48 (4.4%) | 254 (23.5%) | 690 (63.9%) | 160 (14.8%) | 3239 (300.2%) | 1079 |
| **All Ages** | **AD** | 48 (4.6%) | | 23 (2.2%) | 15 (1.4%) | 46 (4.4%) | 79 (7.5%) | 130 (12.4%) | 717 (68.2%) | 187 (17.8%) | 2676 (254.4%) | 1052 |
|  | **FTD** | 155 (19.5%) | | 14 (1.8%) | 20 (2.5%) | 24 (3%) | 107 (13.5%) | 99 (12.5%) | 500 (63%) | 132 (16.6%) | 2262 (284.9%) | 794 |
|  | **Prion** | 82 (27.4%) | | 3 (1%) | 1 (0.3%) | 6 (2%) | 6 (2%) | 35 (11.7%) | 162 (54.2%) | 67 (22.4%) | 999 (334.1%) | 299 |
|  | **DemMot** | 22 (3.4%) | | 5 (0.8%) | 3 (0.5%) | 23 (3.6%) | 32 (5%) | 119 (18.6%) | 387 (60.6%) | 106 (16.6%) | 1750 (273.9%) | 639 |
|  | **Controls** | 2 (0.4%) | | 0 (0%) | 0 (0%) | 5 (1.1%) | 12 (2.6%) | 51 (11.2%) | 321 (70.2%) | 54 (11.8%) | 1447 (316.6%) | 457 |
|  | **Total (% of patients)** | **309 (9.5%)** | | **45 (1.4%)** | **39 (1.2%)** | **104 (3.2%)** | **236 (7.3%)** | **435 (13.4%)** | **2087 (64.4%)** | **546 (16.8%)** | **9134 (281.8%)** | **3241** |

Reference List

1. Lambert MA, Bickel H, Prince M, Fratiglioni L, Von Strauss E, Frydecka D, Kiejna A, Georges J, Reynish EL. Estimating the burden of early onset dementia; systematic review of disease prevalence. Eur J Neurol 2014;21:563-9.

2. Luukkainen L, Bloigu R, Moilanen V, Remes AM. Epidemiology of Frontotemporal Lobar Degeneration in Northern Finland. Dement Geriatr Cogn Dis Extra 2015;5:435-41.

3. Coyle-Gilchrist IT, Dick KM, Patterson K, Vazquez RP, Wehmann E, Wilcox A, Lansdall CJ, Dawson KE, Wiggins J, Mead S, Brayne C, Rowe JB. Prevalence, characteristics, and survival of frontotemporal lobar degeneration syndromes. Neurology 2016.

4. Armstrong RA. Factors determining disease duration in Alzheimer's disease: a postmortem study of 103 cases using the Kaplan-Meier estimator and Cox regression. Biomed Res Int 2014;2014:623487.

5. Onyike CU, Diehl-Schmid J. The epidemiology of frontotemporal dementia. Int Rev Psychiatry 2013;25:130-7.

6. Minikel EV, Vallabh SM, Lek M, Estrada K, Samocha KE, Sathirapongsasuti JF, McLean CY, Tung JY, Yu LP, Gambetti P, Blevins J, Zhang S, Cohen Y, Chen W, Yamada M, Hamaguchi T, Sanjo N, Mizusawa H, Nakamura Y, Kitamoto T, Collins SJ, Boyd A, Will RG, Knight R, Ponto C, Zerr I, Kraus TF, Eigenbrod S, Giese A, Calero M, Pedro-Cuesta J, Haik S, Laplanche JL, Bouaziz-Amar E, Brandel JP, Capellari S, Parchi P, Poleggi A, Ladogana A, O'Donnell-Luria AH, Karczewski KJ, Marshall JL, Boehnke M, Laakso M, Mohlke KL, Kahler A, Chambert K, McCarroll S, Sullivan PF, Hultman CM, Purcell SM, Sklar P, van der Lee SJ, Rozemuller A, Jansen C, Hofman A, Kraaij R, van Rooij JG, Ikram MA, Uitterlinden AG, van Duijn CM, Daly MJ, MacArthur DG. Quantifying prion disease penetrance using large population control cohorts. Sci Transl Med 2016;8:322ra9.

7. Cacace R, Sleegers K, van Broeckhoven C. Molecular genetics of early-onset Alzheimer's disease revisited. Alzheimers Dement 2016;12:733-48.

8. Seltman RE, Matthews BR. Frontotemporal lobar degeneration: epidemiology, pathology, diagnosis and management. CNS Drugs 2012;26:841-70.

9. Ling SC, Polymenidou M, Cleveland DW. Converging Mechanisms in ALS and FTD: Disrupted RNA and Protein Homeostasis. Neuron 2013;79:416-38.

10. Lek M, Karczewski KJ, Minikel EV, Samocha KE, Banks E, Fennell T, O'Donnell-Luria AH, Ware JS, Hill AJ, Cummings BB, Tukiainen T, Birnbaum DP, Kosmicki JA, Duncan LE, Estrada K, Zhao F, Zou J, Pierce-Hoffman E, Berghout J, Cooper DN, Deflaux N, DePristo M, Do R, Flannick J, Fromer M, Gauthier L, Goldstein J, Gupta N, Howrigan D, Kiezun A, Kurki MI, Moonshine AL, Natarajan P, Orozco L, Peloso GM, Poplin R, Rivas MA, Ruano-Rubio V, Rose SA, Ruderfer DM, Shakir K, Stenson PD, Stevens C, Thomas BP, Tiao G, Tusie-Luna MT, Weisburd B, Won HH, Yu D, Altshuler DM, Ardissino D, Boehnke M, Danesh J, Donnelly S, Elosua R, Florez JC, Gabriel SB, Getz G, Glatt SJ, Hultman CM, Kathiresan S, Laakso M, McCarroll S, McCarthy MI, McGovern D, McPherson R, Neale BM, Palotie A, Purcell SM, Saleheen D, Scharf JM, Sklar P, Sullivan PF, Tuomilehto J, Tsuang MT, Watkins HC, Wilson JG, Daly MJ, MacArthur DG. Analysis of protein-coding genetic variation in 60,706 humans. Nature 2016;536:285-91.

11. Cruts M, Theuns J, van Broeckhoven C. Locus-specific mutation databases for neurodegenerative brain diseases. Hum Mutat 2012;33:1340-4.

12. Ryan NS, Nicholas JM, Weston PS, Liang Y, Lashley T, Guerreiro R, Adamson G, Kenny J, Beck J, Chavez-Gutierrez L, De Strooper B, Revesz T, Holton J, Mead S, Rossor MN, Fox NC. Clinical phenotype and genetic associations in autosomal dominant familial Alzheimer's disease: a case series. Lancet Neurol 2016.

13. Lanoiselee HM, Nicolas G, Wallon D, Rovelet-Lecrux A, Lacour M, Rousseau S, Richard AC, Pasquier F, Rollin-Sillaire A, Martinaud O, Quillard-Muraine M, de lS, V, Boutoleau-Bretonniere C, Etcharry-Bouyx F, Chauvire V, Sarazin M, Le B, I, Epelbaum S, Jonveaux T, Rouaud O, Ceccaldi M, Felician O, Godefroy O, Formaglio M, Croisile B, Auriacombe S, Chamard L, Vincent JL, Sauvee M, Marelli-Tosi C, Gabelle A, Ozsancak C, Pariente J, Paquet C, Hannequin D, Campion D. APP, PSEN1, and PSEN2 mutations in early-onset Alzheimer disease: A genetic screening study of familial and sporadic cases. PLoS Med 2017;14:e1002270.
